# Functional interaction of Parkinson's disease-associated LRRK2 with members of the dynamin GTPase superfamily

**DOI:** 10.1093/hmg/ddt600

**Published:** 2013-11-26

**Authors:** Klodjan Stafa, Elpida Tsika, Roger Moser, Alessandra Musso, Liliane Glauser, Amy Jones, Saskia Biskup, Yulan Xiong, Rina Bandopadhyay, Valina L. Dawson, Ted M. Dawson, Darren J. Moore

**Affiliations:** 1 Brain Mind Institute, School of Life Sciences, Ecole Polytechnique Fédérale de Lausanne (EPFL), Lausanne 1015, Switzerland; 2 Neuroregeneration and Stem Cell Programs, Institute for Cell Engineering; 3 Department of Neurology; 4 Department of Neuroscience and; 5 Department of Physiology, The Johns Hopkins University School of Medicine, Baltimore, MD 21205, USA; 6 Adrienne Helis Malvin Medical Research Foundation, New Orleans, LA 70130-2685, USA; 7 Reta Lila Weston Institute of Neurological Studies, University College London Institute of Neurology, London WC1N 1PJ, UK

## Abstract

Mutations in *LRRK2* cause autosomal dominant Parkinson's disease (PD). *LRRK2* encodes a multi-domain protein containing GTPase and kinase domains, and putative protein–protein interaction domains. Familial PD mutations alter the GTPase and kinase activity of LRRK2 *in vitro*. LRRK2 is suggested to regulate a number of cellular pathways although the underlying mechanisms are poorly understood. To explore such mechanisms, it has proved informative to identify LRRK2-interacting proteins, some of which serve as LRRK2 kinase substrates. Here, we identify common interactions of LRRK2 with members of the dynamin GTPase superfamily. LRRK2 interacts with dynamin 1–3 that mediate membrane scission in clathrin-mediated endocytosis and with dynamin-related proteins that mediate mitochondrial fission (Drp1) and fusion (mitofusins and OPA1). LRRK2 partially co-localizes with endosomal dynamin-1 or with mitofusins and OPA1 at mitochondrial membranes. The subcellular distribution and oligomeric complexes of dynamin GTPases are not altered by modulating LRRK2 in mouse brain, whereas mature OPA1 levels are reduced in G2019S PD brains. LRRK2 enhances mitofusin-1 GTP binding, whereas dynamin-1 and OPA1 serve as modest substrates of LRRK2-mediated phosphorylation *in vitro*. While dynamin GTPase orthologs are not required for LRRK2-induced toxicity in yeast, LRRK2 functionally interacts with dynamin-1 and mitofusin-1 in cultured neurons. LRRK2 attenuates neurite shortening induced by dynamin-1 by reducing its levels, whereas LRRK2 rescues impaired neurite outgrowth induced by mitofusin-1 potentially by reversing excessive mitochondrial fusion. Our study elucidates novel functional interactions of LRRK2 with dynamin-superfamily GTPases that implicate LRRK2 in the regulation of membrane dynamics important for endocytosis and mitochondrial morphology.

## INTRODUCTION

Mutations in the *leucine-rich repeat kinase 2* (*LRRK2*, *PARK8*) gene are a common cause of autosomal dominant familial Parkinson's disease (PD) and common variation within the *LRRK2* locus is associated with an increased risk of idiopathic PD (1–3). Despite the major importance of *LRRK2* in the pathogenesis of PD, the biological function of LRRK2 and the mechanisms by which familial mutations precipitate neurodegeneration in PD remain poorly understood (4). The *LRRK2* gene encodes a large multi-domain protein containing a Ras-of-complex (Roc) GTPase domain in tandem with a C-terminal-of-Roc (COR) domain together with a protein kinase domain with similarity to the receptor-interacting protein kinase family (5). LRRK2 additionally contains multiple repeat domains that may mediate protein–protein interactions, including N-terminal LRRK2-specific, ankyrin and leucine-rich and C-terminal WD40 repeat domains. LRRK2 displays GTPase and kinase activity *in vitro* and PD-associated mutations can either enhance kinase activity (G2019S) or impair GTP hydrolysis activity (R1441C/G/H, Y1699C) (6–14). Although familial mutations have divergent effects on LRRK2 enzymatic activity, the overexpression of LRRK2 familial mutants commonly enhances neuronal toxicity including inhibition of neurite outgrowth and induction of apoptotic cell death (4,7,13,15–17). The mechanisms regulating the neurotoxic actions of LRRK2 are not fully understood although kinase activity is required for the pathogenic effects of the G2019S mutation in cultured neurons and in rodents (7,16,18,19). GTPase activity also contributes to LRRK2-dependent neuronal toxicity (14,16,20–23).

In attempting to explain the physiological function and neurotoxic actions of LRRK2, a number of cellular pathways, processes and/or organelles have been implicated (4). LRRK2 has been shown to regulate synaptic vesicle trafficking (24,25), endocytosis and exocytosis (14,25,26), Golgi complex integrity (21,27), the actin cytoskeleton and microtubule networks (28,29), the autophagy–lysosomal pathway (14,30–33), protein sorting and translation (34,35) and mitochondrial morphology and activity (36–38) through as yet unclear mechanisms. The seemingly diverse effects regulated by LRRK2 in mammalian cells are supported by its broad subcellular distribution with enrichment of LRRK2 upon multiple intracellular membranous and vesicular structures including endosomes, lysosomes, mitochondria, microtubule transport vesicles, lipid rafts, the Golgi complex, the endoplasmic reticulum and synaptosomes (24,39,40). Such a widespread distribution of LRRK2 could suggest a general housekeeping function in regulating membrane biogenesis, dynamics and/or trafficking within neurons.

To explore the molecular basis for the normal and pathogenic actions of LRRK2 in neurons, it has been informative to identify interacting proteins and kinase substrates since the domain structure of LRRK2 implies that it most likely functions as a complex protein scaffold to regulate cellular signaling pathways in a kinase- and/or GTPase-dependent manner (4). Although a number of putative substrates of LRRK2 kinase activity have so far been identified *in vitro* and in invertebrate models (9,21,22,25,28,41,42), validation of LRRK2-mediated substrate phosphorylation in mammalian cells is presently lacking (43,44). LRRK2-interacting proteins have also been identified that have provided important insight into the regulation of LRRK2 GTPase activity (i.e. ARHGEF7 and ArfGAP1) (21,22,45), stability/metabolism (i.e. CHIP and Hsp90) (46,47) and subcellular localization (i.e. 14-3-3 proteins) (48). Interacting proteins also provide a basis for the observed effects of LRRK2 on microtubule network dynamics (i.e. β-tubulin) (28), actin cytoskeleton dynamics (i.e. moesin) (29), mitochondrial dynamics (i.e. Drp1) (49,50) and synaptic vesicle mobility and endocytosis (i.e. Rab5b and endophilin A, etc.) (25,26). The detailed biochemical and cellular characterization of such protein–protein interactions is important for understanding the full repertoire of actions exerted by LRRK2 in mammalian cells under normal and pathological conditions.

Here, we identify and functionally characterize the novel interaction of LRRK2 with multiple members of the dynamin superfamily of large GTPases, including proteins regulating endocytosis and mitochondrial dynamics. Our data implicate LRRK2 in the regulation of membrane dynamics important for endocytosis and mitochondrial morphology through a common interaction with dynamin-superfamily GTPases. Moreover, we describe a useful pipeline of LRRK2-related assays that can be employed for the rigorous functional validation of LRRK2-interacting proteins.

## RESULTS

### LRRK2 commonly interacts with members of the dynamin GTPase superfamily

**Figure 1.**
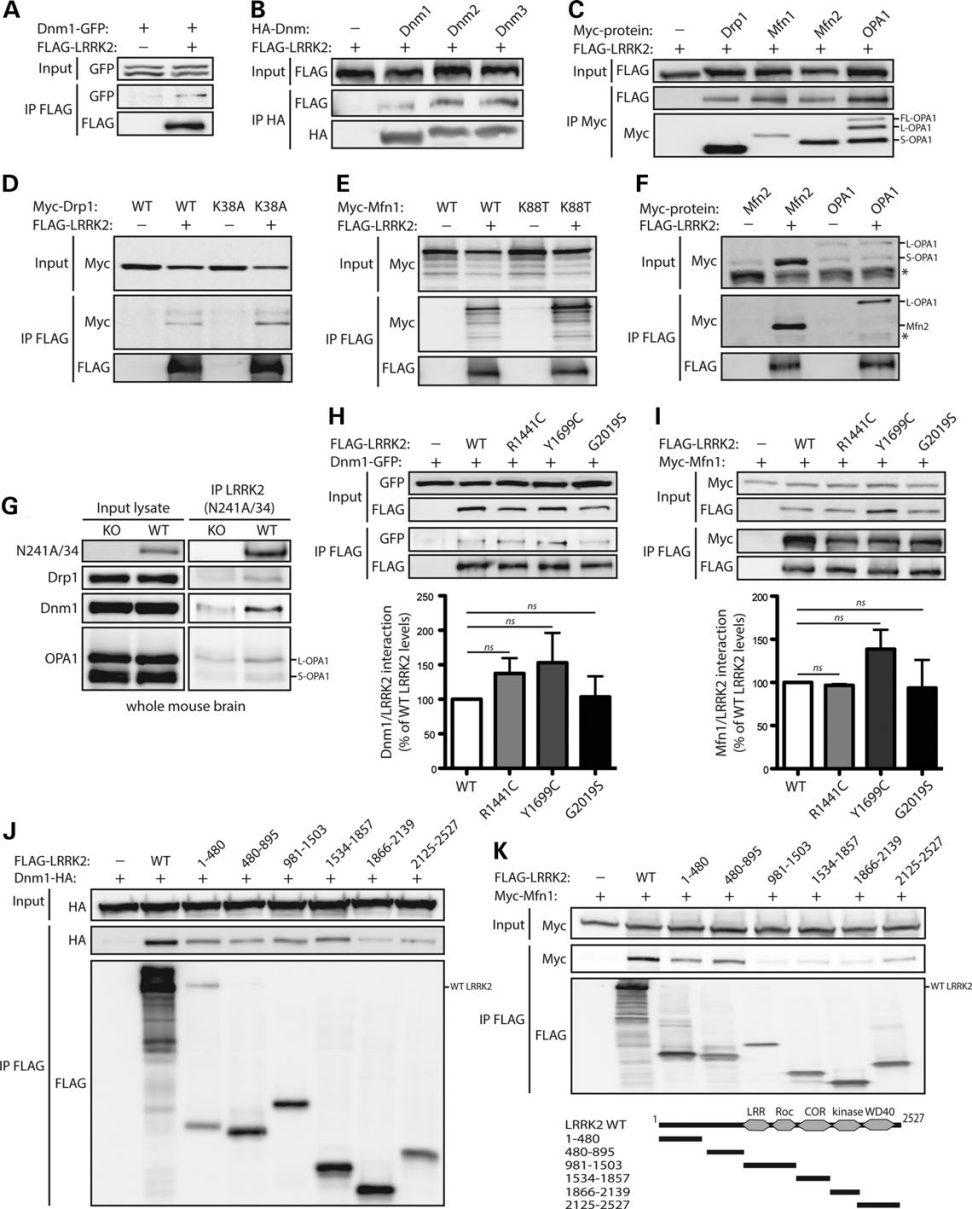
LRRK2 commonly interacts with members of the dynamin GTPase superfamily. (**A**) FLAG-tagged human LRRK2 interacts with GFP-tagged human Dnm1 following immunoprecipitation (IP) with anti-FLAG antibody from HEK-293T cells. (**B**) HA-tagged mouse Dnm1, Dnm2 and Dnm3 interact with FLAG-LRRK2 following IP with anti-HA antibody. (**C**) Myc-tagged Drp1, Mfn1, Mfn2 and OPA1 interact with FLAG-LRRK2 following IP with anti-myc antibody. (**D–F**) FLAG-LRRK2 interacts with (D) Myc-tagged WT and K38A Drp1, (E) WT and K88T Mfn1 and (F) Mfn2 and OPA1, following IP with anti-FLAG antibody from HEK-293T cells. (**G**) LRRK2 interacts with Drp1, Dnm1 and OPA1 in whole brain extracts from WT mice but not LRRK2 knockout (KO) mice following IP with anti-LRRK2 antibody (clone N241A/34). Data are representative of two-independent experiments. (**H**) Dnm1-GFP and (**I**) Myc-Mfn1 interact with WT and PD-associated mutant forms of FLAG-LRRK2 following IP with anti-FLAG antibody from HEK-293T cells. Densitometric analysis reveals no significant differences in the interactions of Dnm1 or Mfn1 with R1441C, Y1699C and G2019S LRRK2 compared with WT LRRK2. Data represent the level of interaction of Dnm1 or Mfn1 with LRRK2 expressed as a percent of the interaction with WT LRRK2. The levels of Dnm1 or Mfn1 IP were first normalized to their respective input levels, and then further normalized to LRRK2 IP levels. Bars represent the mean ± SEM (*n* = 3 experiments). n.s., non-significant. (**J** and **K**) Domain mapping reveals the interaction of HA-Dnm1 with full-length (WT) LRRK2, residues 1–480, 480–895, 981–1503 and 1534–1857 of LRRK2, and to a lesser extent other LRRK2 domains (J), whereas Myc-Mfn1 interacts with full-length LRRK2 and residues 1–480, 480–895 and 2125–2527 of LRRK2 (K) following IP with anti-FLAG antibody. (K) Domain organization of LRRK2 deletion mutants is indicated. Data are representative of at least three-independent experiments.

To identify novel interacting proteins for LRRK2, we performed a yeast two-hybrid screen using an adult human brain cDNA library as prey with the N-terminal region (residues 1–500) of human LRRK2 as bait. We identified human dynamin-1 (Dnm1) as a putative interacting partner of LRRK2 (data not shown). Dnm1 has recently been identified as a putative LRRK2-interacting protein in a mass spectrometry-based screen of brain synaptosomes, although verification of this interaction was not provided (24). Dnm1 is an 864 amino acid neuronal-specific GTPase that plays an important role in regulating clathrin-mediated endocytosis by mediating membrane scission events (51–53). We and others have previously described a role for LRRK2 in regulating synaptic vesicle endocytosis and exocytosis and thus we elected to further explore the potential relationship between LRRK2 and Dnm1 (14,24,26). The interaction of these two proteins was first assessed by co-immunoprecipitation (co-IP) analysis in HEK-293T cells exogenously expressing FLAG-tagged LRRK2 and GFP-tagged Dnm1. Following IP of full-length LRRK2, we detect a clear interaction with full-length Dnm1 (Fig. 1A). Dnm1 belongs to the dynamin superfamily of large GTPases whose members notably include classical dynamins (i.e. Dnm1, Dnm2 and Dnm3) and dynamin-related proteins (i.e. dynamin-related protein 1 [Drp1], optic atrophy protein 1 [OPA1] and mitofusin [Mfn] protein families) (52), whereas classical dynamins mediate vesicle scission events at the plasma membrane and *trans*-Golgi network to regulate endocytosis, dynamin-related proteins are involved in mitochondrial membrane fission (i.e. Drp1) and fusion (i.e. Mfn1, Mfn2 and OPA1) events to regulate mitochondrial dynamics (52–54). To determine whether LRRK2 broadly interacts with members of the dynamin superfamily, we compared the interaction of LRRK2 with the classical dynamins, Dnm1, Dnm2 and Dnm3, by co-IP in HEK-293T cells expressing FLAG-tagged LRRK2 and full-length HA-tagged Dnm1-3. We detect a common interaction of LRRK2 with Dnm1, Dnm2 and Dnm3 (Fig. 1B).

LRRK2 has also been implicated in regulating mitochondrial activity and morphology (36,37,49,50). Accordingly, we asked whether LRRK2 interacts with the dynamin-related proteins, Drp1, Mfn1, Mfn2 and OPA1. The interaction of LRRK2 with dynamin-related proteins was assessed by co-IP in HEK-293T cells expressing FLAG-tagged LRRK2 and Myc-tagged Drp1, Mfn1, Mfn2 or OPA1. Following IP of full-length Drp1, Mfn1, Mfn2 and OPA1, we detect a robust interaction with full-length LRRK2 (Fig. 1C). In the reverse experiment, IP of full-length LRRK2 reveals an interaction with Drp1, Mfn1, Mfn2 and OPA1 (Fig. 1D–F). Disruption of the GDP/GTP-binding capacity of Drp1 and Mfn1 by mutating a critical lysine residue in the conserved guanine nucleotide phosphate-binding motif (K38A in Drp1 and K88T in Mfn1) does not alter the degree of interaction with LRRK2 (Fig. 1D and E). Expression of full-length OPA1 gives rise to an unprocessed full-length form (FL-OPA1) and processed large (L-OPA1) and short (S-OPA1) mature forms by sequential N-terminal proteolytic cleavage of a mitochondrial-targeting sequence (MTS) and an adjacent transmembrane helix, respectively (Fig. 1C) (55). LRRK2 selectively interacts with the mature L-OPA1 form lacking the MTS (Fig. 1F), which is known to be anchored to the mitochondrial inner membrane via its N-terminal transmembrane helix (55). We next sought to verify the interaction of LRRK2 and dynamin GTPases *in vivo*. LRRK2 interacts with Drp1, Dnm1 and OPA1 (particularly L-OPA1) in whole brain extracts derived from wild-type (WT) mice following immunoprecipitation with a LRRK2-specific monoclonal antibody (N241A/34), whereas Drp1, Dnm1 and OPA1 are immunoprecipitated to a lesser or negligible extent in extracts derived from LRRK2 knockout mice (Fig. 1G). An interaction of LRRK2 with Mfn2 could not be demonstrated, whereas we are not able to reliably detect Mfn1 in mouse brain using currently available antibodies (data not shown).

To explore the effects of PD-associated mutations on the interaction of LRRK2 with Dnm1, Mfn1 and OPA1, we conducted co-IP experiments in HEK-293T cells expressing FLAG-tagged LRRK2 variants with either GFP-tagged Dnm1 or Myc-tagged Mfn1 and OPA1. Compared with WT LRRK2, the familial PD mutations R1441C, Y1699C and G2019S do not significantly alter the interaction of LRRK2 with Dnm1 (Fig. 1H), Mfn1 (Fig. 1I) or OPA1 (Supplementary Material, Fig. S1B). To identify the domain of LRRK2 that interacts with selected dynamin GTPases, co-IP assays were conducted in cells expressing various deletion mutants of LRRK2 together with HA-tagged Dnm1 or Myc-tagged Mfn1 and OPA1. Following IP of LRRK2 deletion mutants we find that Dnm1 modestly interacts with multiple LRRK2 domains, with particular enrichment for residues 1–480 (containing LRRK2-specific and armadillo repeats), 480–895 (containing ankyrin repeats), 895–1503 (containing LRR and Roc GTPase domains) and 1534–1857 (containing the COR domain) of LRRK2 (Fig. 1J). OPA1 preferentially interacts with full-length LRRK2 but not with isolated domains of LRRK2 (Supplementary Material, Fig. S1A), suggesting that the intact full-length protein is required for this interaction. Mfn1 interacts selectively with N-terminal (residues 1–480 and 480–895) and C-terminal (residues 2125–2527 containing WD40 repeats) regions of LRRK2 (Fig. 1K), known to mediate protein–protein interactions. Collectively, these data reveal that LRRK2 commonly interacts with members of the dynamin superfamily in mammalian cells and brain.

### LRRK2 co-localizes with dynamin-1 in endosomal compartments

**Figure 2.**
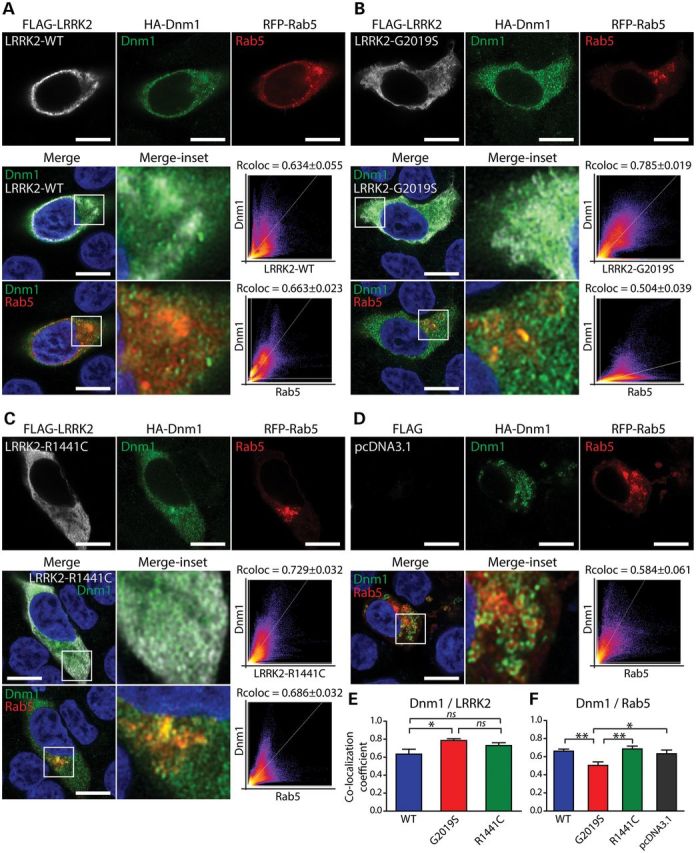
LRRK2 partially co-localizes with Dnm1 at early endosomes in neural cells. (**A–C**) Confocal fluorescence microscopy reveals the partial co-localization of FLAG-tagged human LRRK2 (WT, G2019S and R1441C) with HA-tagged human Dnm1 at RFP-Rab5-positive early endosomal vesicles in human SH-SY5Y neural cells. Cytofluorograms and co-localization coefficients (Rcoloc; mean ± SEM, *n* = 10–12 cells/condition) reveal the extent of co-localization between LRRK2 and Dnm1 fluorescence signals. (A–**D**) Confocal images, cytofluorograms and co-localization coefficients revealing the effect of overexpressing human LRRK2 variants on the degree of co-localization of Dnm1 and Rab5, relative to control cells transfected with empty vector (pcDNA3.1). Confocal images are taken from a single *z*-plane at 0.1 µm thickness. Images are representative of multiple cells from at least two-independent transfection experiments. Scale bars: 10 µm. (**E** and **F**) Graphs indicating co-localization coefficients (mean ± SEM, *n* = 10–12 cells) for (E) Dnm1 and LRRK2 variants and (F) Dnm1 and Rab5. The G2019S mutation significantly increases the co-localization of LRRK2 with Dnm1 (E), and reduces the co-localization of Dnm1 with Rab5-positive endosomes (F). *P < 0.05 and **P < 0.01 by one-way ANOVA with Newman–Keuls *post hoc* analysis, as indicated. n.s., non-significant.

To further characterize the interaction of LRRK2 with dynamin GTPases, and where in cells these interactions might occur, we assessed their co-localization in mammalian cells by immunocytochemistry and confocal microscopy. We have previously shown that LRRK2 partly localizes to clathrin-coated endosomes by electron microscopy and is enriched within microsomal and synaptosomal membranes by subcellular fractionation in rodent brain (39). Since Dnm1 is known to localize to early endosomes where it regulates clathrin-mediated endocytosis (52), we first sought to determine which endosomal compartments co-localize with LRRK2. Co-expression of FLAG-LRRK2 with RFP-Rab5 and GFP-Rab7 in rat primary cortical neurons reveals a high degree of localization of LRRK2 with Rab5-positive early endosomes (Rcoloc = 0.79 ± 0.04) but a smaller degree of co-localization with Rab7-positive late endosomes (Rcoloc = 0.41 ± 0.06) (Supplementary Material, Fig. S2). We compared the effects of familial PD mutations on the co-localization of LRRK2 with Rab5 and Rab7 but do not observe significant differences between WT, R1441C and G2019S variants (Supplementary Material, Fig. S2). These data indicate that LRRK2 preferentially co-localizes with early endosomes, a compartment where Dnm1 is known to prominently reside. Next, we co-expressed FLAG-LRRK2 variants and RFP-Rab5 together with HA-tagged Dnm1 plasmids in human SH-SY5Y neural cells to assess their co-localization. We could observe the equivalent co-localization of WT and R1441C LRRK2 with Dnm1 (Rcoloc: 0.634 ± 0.055 for WT or 0.729 ± 0.032 for R1441C) with significantly increased co-localization of Dnm1 with the G2019S variant (Rcoloc: 0.785 ± 0.019). We also observe marked co-localization of Dnm1 with Rab5-positive early endosomes in the presence or absence of LRRK2 overexpression (Rcoloc: 0.584 ± 0.061 for control vector; 0.663 ± 0.023 for WT LRRK2; 0.686 ± 0.032 for R1441C LRRK) but with significantly reduced co-localization due to G2019S LRRK2 (Rcoloc: 0.504 ± 0.039) (Fig. 2). Taken together, these data indicate the partial co-localization of LRRK2 and Dnm1 in or adjacent to early endosomal compartments, suggesting that these proteins most likely interact at the cytoplasmic face of endosomal vesicles. The familial G2019S mutation increases the co-localization of LRRK2 with Dnm1 but reduces the proportion of Dnm1 on early endosomes.

### LRRK2 co-localizes with dynamin-related GTPases at mitochondrial membranes

**Figure 3.**
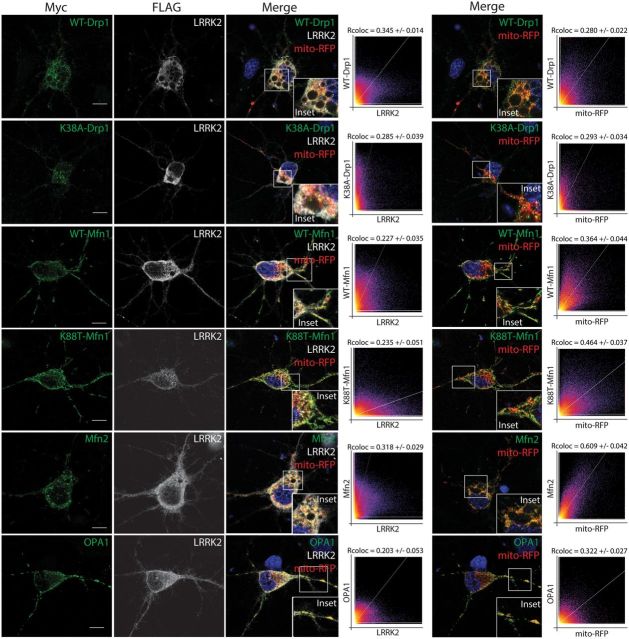
LRRK2 partially co-localizes with dynamin-related GTPases at mitochondria in cortical neurons. Confocal fluorescence microscopy reveals the partial co-localization of FLAG-tagged human LRRK2 with Myc-tagged Drp1 (WT and K38A), Mfn1 (WT and K88T), Mfn2 and OPA1 at mito-RFP-positive mitochondria in rat primary cortical neurons. Cytofluorograms and co-localization coefficients (Rcoloc; mean ± SEM, *n* ≥ 5 cells) reveal the extent of co-localization of LRRK2 with Drp1, Mfn1, Mfn2 or OPA1 fluorescence signals. The degree of co-localization of Drp1, Mfn1, Mfn2 and OPA1 with mito-RFP fluorescence signals is also indicated for comparison. Confocal images are taken from a single *z*-plane at 0.1 µm thickness. Images are representative of multiple neurons from at least two-independent transfection experiments. Scale bars: 10 µm.

We have previously shown that LRRK2 localizes in part to the mitochondrial outer membrane and matrix compartment in mammalian cells and rodent brain by confocal microscopy, submitochondrial fractionation and electron microscopy (12,39). To explore the co-localization of LRRK2 with dynamin-related GTPases, we co-expressed FLAG-LRRK2 and Myc-tagged Drp1, Mfn1, Mfn2 and OPA1 together with the mitochondrial marker, mito-RFP, in primary cortical neurons. Drp1 normally localizes diffusely within the cytoplasm but can translocate to the mitochondrial outer membrane regulated by posttranslational modifications to mediate mitochondrial fission (56). LRRK2 partially co-localizes with WT and K38A Drp1 (Rcoloc: 0.345 ± 0.014 for WT or 0.285 ± 0.039 for K38A Drp1) mostly within the cytoplasm but to a small extent upon RFP-positive mitochondria in neurons (Fig. 3). Mfn1 and Mfn2 are localized to the outer membrane of mitochondria where they regulate outer membrane fusion, whereas OPA1 is associated with the mitochondrial inner membrane and intermembrane space and regulates fusion of the inner membrane (54). LRRK2 partially co-localizes with WT and K88T Mfn1 (Rcoloc: 0.227 ± 0.035 for WT or 0.235 ± 0.051 for K88T Mfn1), Mfn2 (Rcoloc: 0.318 ± 0.029) and OPA1 (Rcoloc: 0.203 ± 0.053) upon RFP-positive mitochondria (Fig. 3). As expected, Mfn1, Mfn2 and OPA1 localization is enriched upon or in close proximity to mito-RFP-positive mitochondria (Fig. 3), consistent with their known association with mitochondrial membranes. Collectively, these data indicate the partial co-localization of LRRK2 with dynamin-related GTPases and suggest that LRRK2 and Drp1 most likely interact within the cytoplasm whereas LRRK2 and Mfn1, Mfn2 and OPA1 interact at or adjacent to mitochondrial membranes.

### Impact of LRRK2 expression on subcellular distribution and native complexes of dynamin GTPases in mouse brain

**Figure 4.**
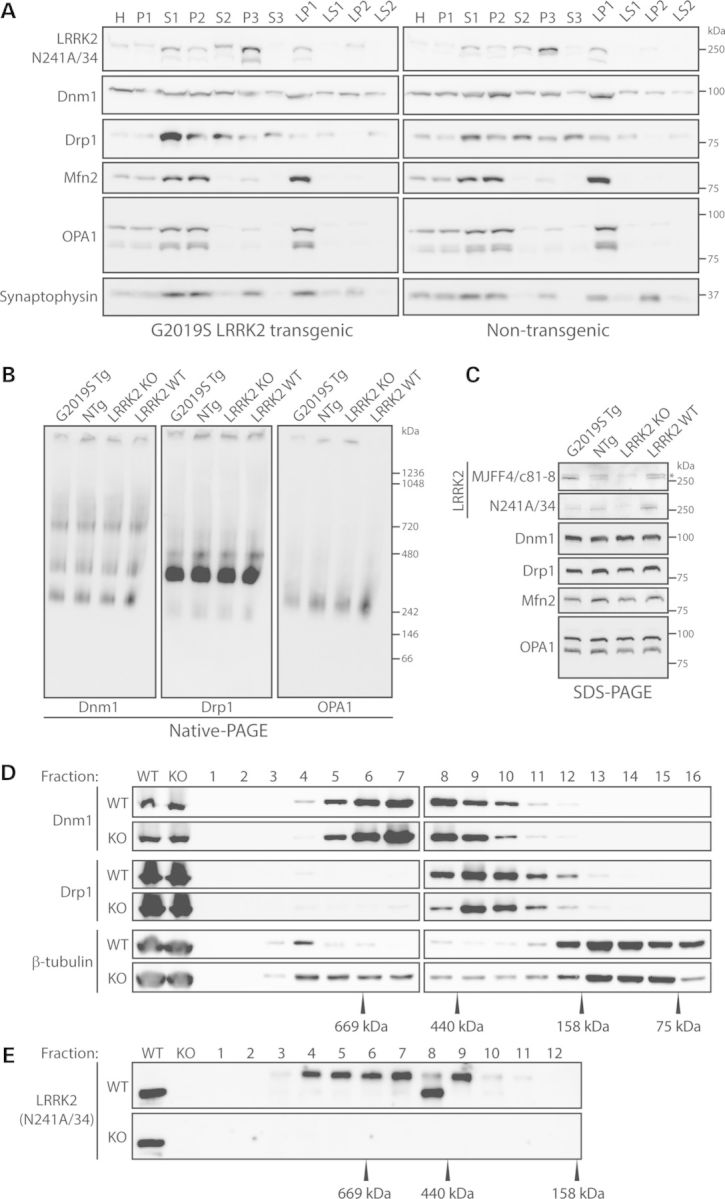
Subcellular distribution and native complexes of dynamin GTPases are not altered by LRRK2 in mouse brain. (**A**) Subcellular fractionation of cerebral cortex tissue derived from human G2019S LRRK2 transgenic and non-transgenic mice. Dnm1 and Drp1 are broadly distributed across multiple membrane and soluble fractions, whereas Mfn2 and OPA1 are enriched in heavy membrane (P2) and synaptosomal membrane (LP1) fractions. LRRK2 is broadly detected with enrichment in light membrane/microsomal (P3), synaptosomal LP1 and synaptic vesicle-enriched (LP2) membrane fractions. The distribution of the synaptic vesicle-associated protein, synaptophysin 1, demonstrates enrichment of membranes in P2, P3, LP1 and LP2 fractions, whereas Mfn2 and OPA1 indicate enrichment of mitochondria in P2 and LP1 fractions. (**B**) Native-PAGE and (**C**) SDS–PAGE analysis of equivalent cerebral cortex extracts derived from human G2019S LRRK2 transgenic (Tg) and non-transgenic (NTg) mice, and LRRK2 KO and WT mice, revealing similar oligomeric protein complexes for Dnm1, Drp1 and OPA1. (C) LRRK2 antibodies confirm the absence of LRRK2 in KO mice (mouse-selective N241A/34 antibody) and human G2019S LRRK2 expression in transgenic mice (human-selective MJFF4/c81-8 antibody; lower band = LRRK2; asterisk indicates non-specific upper band). (**D** and **E**) Size-exclusion chromatography on soluble whole brain extracts from WT and LRRK2 KO mice. Sequential fractions (#1–16, 0.5 ml) and total homogenates (WT or KO) were analyzed by western blotting with antibodies to Dnm1, Drp1 and LRRK2 (N241A/34), or β-tubulin as a loading control. The elution profile of Dnm1 and Drp1 is similar in WT and KO brains, whereas the elution profile of individual standards is indicated. LRRK2 antibody (N241A/34) confirms the absence of LRRK2 in KO mice. Blots are representative of duplicate experiments. Molecular mass markers are indicated in kDa.

To begin to explore the potential functional interaction of LRRK2 with dynamin GTPases, we examined the impact of the common G2019S mutation in LRRK2 on the subcellular distribution of endogenous dynamin GTPases in adult mouse brain. Subcellular fractionation was conducted on cerebral cortex tissue derived from human G2019S LRRK2 transgenic and non-transgenic mice (Fig. 4A). Dnm1 is broadly distributed across all fractions but with enrichment in the heavy membrane (P2), light membrane/microsomal (P3) and synaptosomal membrane (LP1) fractions. Drp1 is also widely distributed across soluble and membrane fractions with enrichment in the soluble S1, S2 and S3 fractions (Fig. 4A). Mfn2 and OPA1 are enriched within the heavy membrane/mitochondrial (P2), synaptosomal membrane (LP1) and soluble S1 fractions (Fig. 4A). For comparison, LRRK2 is enriched in the light membrane/microsomal (P3) fraction and is detected in the synaptosomal (LP1), synaptic vesicle (LP2) and heavy (P2) membrane fractions and soluble (S1 and S2) fractions (Fig. 4A). LRRK2 co-fractionates with Dnm1, Drp1, Mfn2 and OPA1 in the synaptosomal (LP1) and heavy (P2) membrane fractions, and with Dnm1 and Drp1 in the light membrane (P3) fraction (Fig. 4A) of mouse brain. However, the subcellular fractionation profiles of Dnm1, Drp1, Mfn2 and OPA1 in mouse brain are not altered in human G2019S LRRK2 transgenic mice compared with their non-transgenic littermates (Fig. 4A). We are unable to reliably assess the subcellular distribution of Mfn1 in these mouse brain extracts due to a lack of sufficiently specific antibodies for detecting endogenous Mfn1. These data reveal that the pathogenic G2019S mutation in human LRRK2 does not influence the subcellular distribution of Dnm1, Drp1, Mfn2 and OPA1 in the adult mouse brain.

Dynamin GTPases are known to function through the formation of homo-oligomeric complexes to mediate membrane fission or fusion (52,54). To explore the impact of LRRK2 expression on the formation of oligomeric complexes by dynamin GTPases, we conducted native-PAGE analysis on soluble extracts of cerebral cortex derived from G2019S LRRK2 transgenic and non-transgenic mice, and LRRK2 knockout (KO) mice and their WT littermates (Fig. 4B). Dnm1 forms a broad range of native complexes from ∼300 kDa to ∼1 mDa with distinct complexes at ∼300, ∼480 and ∼800 kDa consistent with a range of oligomeric species. Drp1 forms a major native complex of 350–480 kDa in addition to less abundant complexes. OPA1 also forms a broad range of native complexes from ∼300 to ∼720 kDa but with a distinct complex centered at 300–350 kDa. Importantly, these native brain complexes are not altered by the transgenic overexpression of human G2019S LRRK2 or by LRRK2 deletion in KO mice compared with control mice (Fig. 4B). We are not able to reliably detect Mfn2 complexes by native PAGE in these soluble brain extracts (data not shown). SDS–PAGE analysis conducted in parallel on these soluble mouse brain extracts reveals that the steady-state levels of Dnm1, Drp1, Mfn2 and OPA1 are not altered by modulating LRRK2 expression (Fig. 4C). To further explore oligomeric complexes of dynamin GTPases, we conducted size-exclusion chromatography on soluble brain extracts derived from adult WT and LRRK2 KO mice (Fig. 4D and E). Dnm1 elutes over a broad range (fractions 4–12) with the majority of signal between 440 and 669 kDa compatible with an oligomeric complex (Fig. 4D). Drp1 elutes over a smaller range (fractions 8–13) with the majority of signal between 158 and 440 kDa (Fig. 4D). For comparison, LRRK2 broadly elutes within fractions 3–11 with the highest signal centered at 669 kDa compatible with a dimer-sized LRRK2 complex (Fig. 4E). Importantly, the hydrodynamic volumes of Dnm1 and Drp1 under native conditions are not altered by the absence of LRRK2 expression in KO mice (Fig. 4D). We are not able to reliably detect Mfn2 or OPA1 complexes by size-exclusion chromatography in these soluble brain extracts (data not shown). Collectively, these data indicate that LRRK2 expression does not influence the formation of endogenous Dnm1, Drp1 or OPA1 native protein complexes or the total levels of these proteins in the mouse brain.

### Reduced levels of mature OPA1 in G2019S PD brains

**Figure 5.**
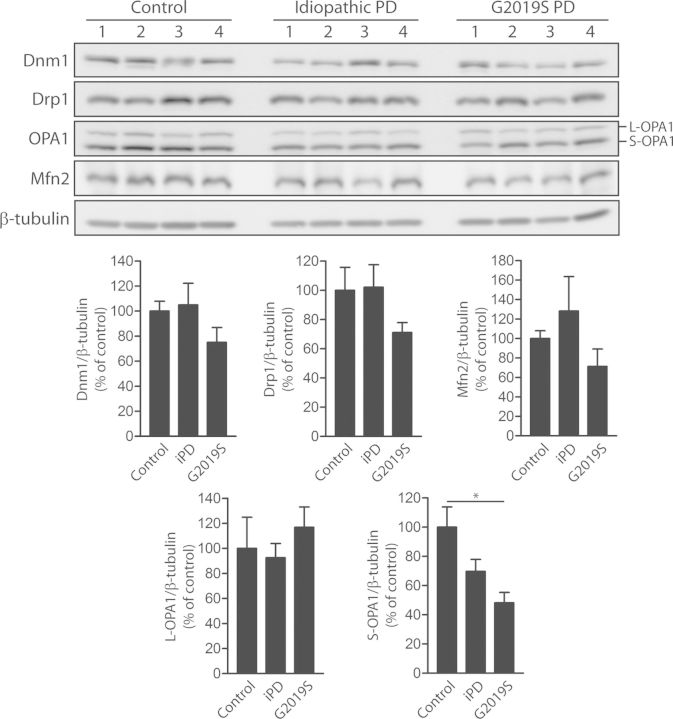
Reduced levels of mature S-OPA1 in G2019S mutant PD brains. Western blot analysis of frontal cortex soluble fractions from human control, idiopathic PD (iPD) or G2019S LRRK2 PD subjects with antibodies to Dnm1, Drp1, OPA1 and Mfn2, or β-tubulin as a protein loading control. Densitometric analysis of Dnm1, Drp1, OPA1 (L-OPA1 and S-OPA1) and Mfn2 in idiopathic or G2019S PD brains compared with control brains are indicated. The levels of each protein were normalized to β-tubulin levels, and expressed as a percent of control subjects (mean ± SEM, *n* = 4 subjects/group). *P < 0.05 by one-way ANOVA with Newman–Keuls *post hoc* analysis, as indicated.

We next sought to determine the impact of G2019S LRRK2 expression on the steady-state levels of Dnm1, Drp1, Mfn2 and OPA1 in the human brain. Accordingly, soluble extracts derived from the frontal cortex of G2019S mutant or idiopathic PD brains and normal control brains were subjected to western blot analysis for each protein (Fig. 5). We observe no significant differences in the steady-state levels of Dnm1, Drp1 and Mfn2 in G2019S mutant or idiopathic PD brains compared with control brains (Fig. 5). However, we observe a significant marked reduction of the mature short form of OPA1 (S-OPA1) but not the long form (L-OPA1) in G2019S mutant PD brains compared with control brains, and a non-significant reduction of S-OPA1 in idiopathic PD brains (Fig. 5). Our data demonstrate that the pathogenic G2019S mutation selectively reduces the steady-state levels of the mature short form of OPA1 in the human frontal cortex.

### LRRK2 enhances the levels of GTP-bound mitofusin-1

**Figure 6.**
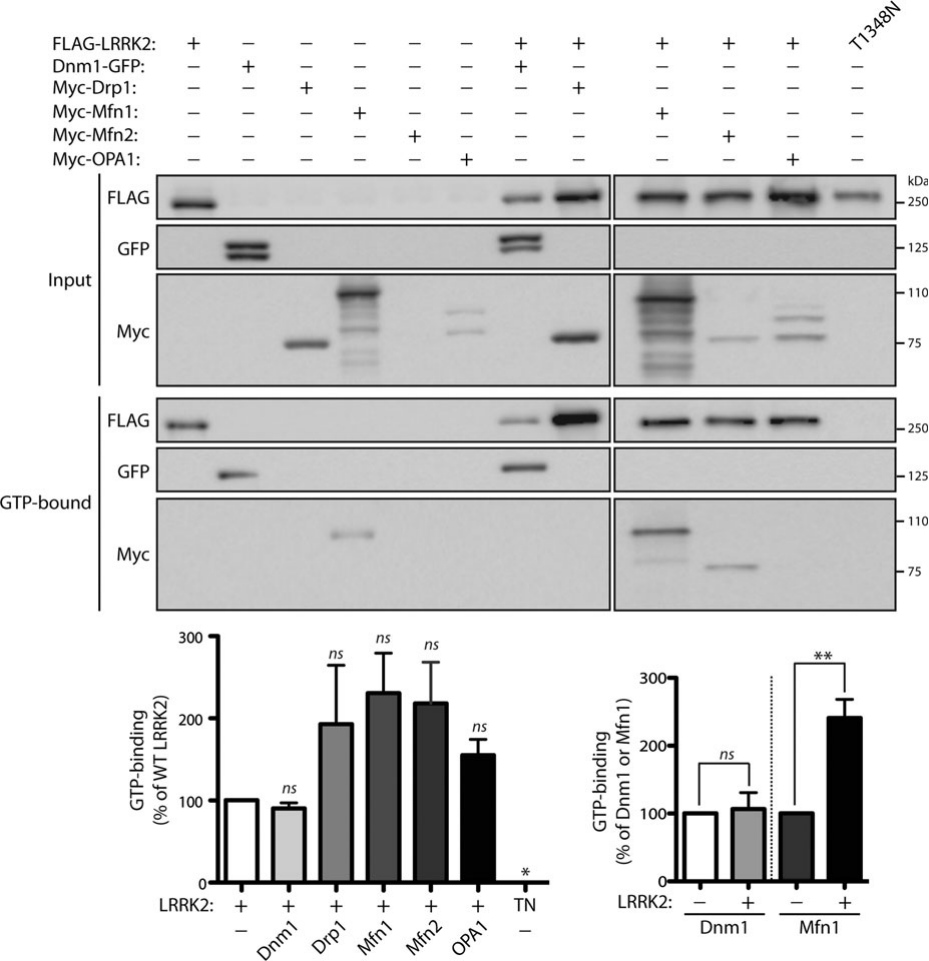
LRRK2 enhances the levels of GTP-bound Mfn1. Dynamin GTPases (Dnm1, Drp1, Mfn1, Mfn2 and OPA1) fail to significantly influence the steady-state levels of FLAG-tagged WT LRRK2 bound to GTP following pull-down assays with GTP-agarose from HEK-293T cell extracts. The specificity of LRRK2 GTP binding is indicated by the absence of binding of the GDP/GTP-binding-deficient LRRK2 mutant, T1348N (TN). Densitometric analysis reveals significantly enhanced steady-state levels of Mfn1 but not Dnm1 bound to GTP in the presence of LRRK2. Note that only Mfn1 and Dnm1 reveal detectable GTP binding when expressed alone compared with Drp1, Mfn2 and OPA1. Molecular mass markers are indicated in kDa. Data represent the level of LRRK2 (left) or Dnm1 and Mfn1 (right) GTP binding expressed as a percent of the levels of each protein alone. GTP-bound protein levels were normalized to input protein levels. Bars represent the mean ± SEM (*n* = 3 experiments). *P < 0.05 or **P < 0.002 by one-way ANOVA with Newman–Keuls *post hoc* analysis, as indicated. n.s., non-significant.

To determine whether the interaction with LRRK2 may serve to regulate the GTPase activity of dynamin-superfamily members, and *vice versa*, we explored the effects of co-expressing LRRK2 and dynamin GTPases on their capacity to bind GTP. To monitor the steady-state levels of GTP-bound LRRK2 or dynamin GTPases, we conducted pull-down assays using GTP-agarose from HEK-293T cell extracts expressing each protein alone or together. Co-expression with Drp1, Mfn1, Mfn2 or OPA1, but not with Dnm1, results in a marked yet non-significant increase in the levels of GTP-bound LRRK2 (Fig. 6). Control experiments confirm the specificity of LRRK2 binding to immobilized GTP as the T1348N variant of LRRK2 abolishes binding to GTP (Fig. 6). Of the five dynamin GTPases examined, only Dnm1 and Mfn1 consistently bind to GTP at detectable levels in this assay when expressed alone (Fig. 6). Co-expression with LRRK2 fails to alter the levels of GTP-bound Dnm1, but results in a marked and significant increase in GTP-bound Mfn1 (Fig. 6). Since both LRRK2 and dynamin GTPases display GTP hydrolysis activity, it was not possible to discriminate their specific functional effects on each other when incubated together in *in vitro* assays measuring the hydrolysis of GTP to GDP (data not shown). Collectively, our data suggest that dynamin-related GTPases enhance the GTP-binding capacity of LRRK2, whereas oppositely LRRK2 markedly enhances the GTP-binding activity of Mfn1.

### Dynamin GTPases are modest substrates of LRRK2-mediated phosphorylation *in vitro*

**Figure 7.**
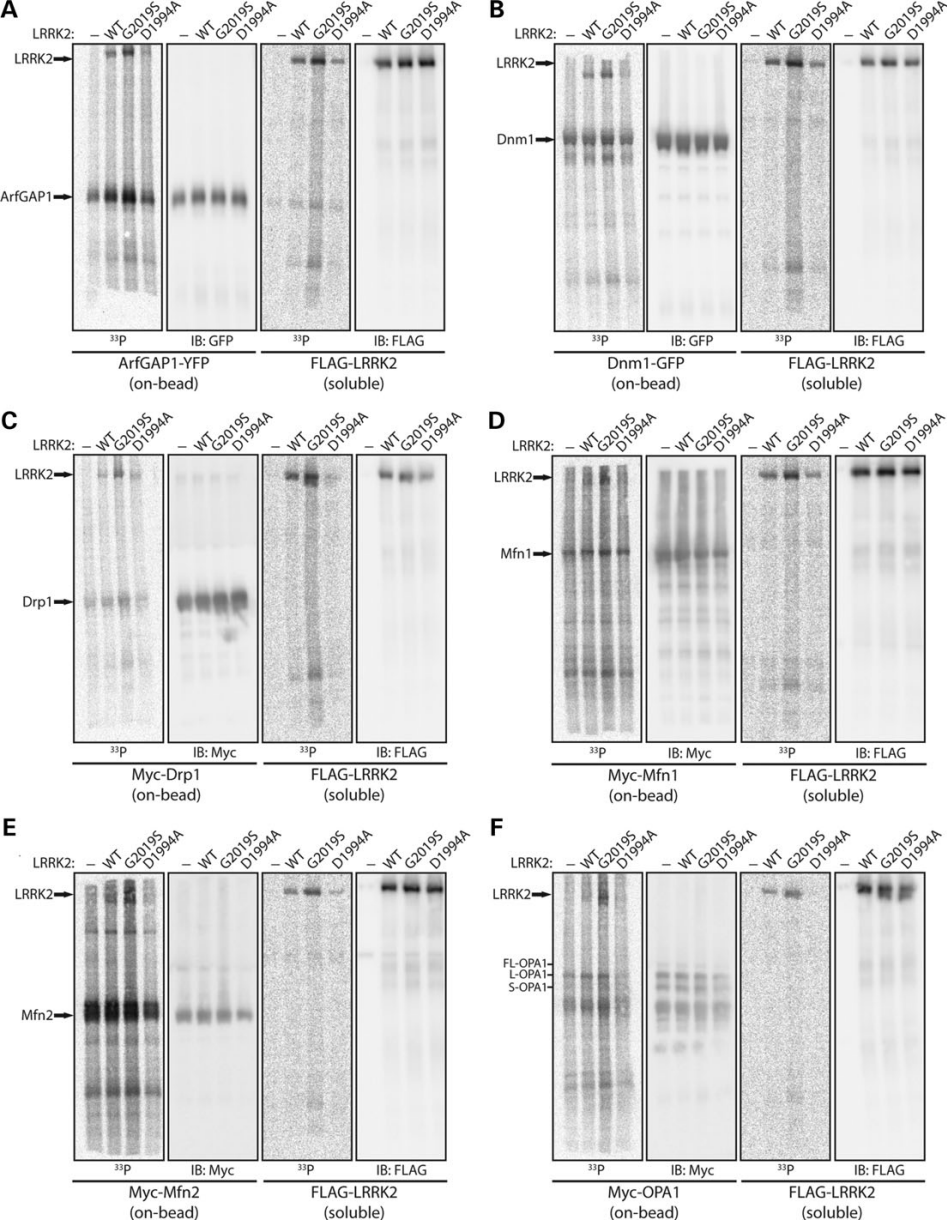
Dynamin GTPases are modestly phosphorylated by LRRK2 *in vitro. In vitro* kinase assays with [^33^P]-γ-ATP, soluble recombinant full-length FLAG-tagged LRRK2 variants (WT, G2019S or D1994A) and immunopurified ‘on-bead’ YFP-tagged ArfGAP1 (**A**), GFP-tagged Dnm1 (**B**), and Myc-tagged Drp1 (**C**), Mfn1 (**D**), Mfn2 (**E**) or OPA1 (**F**), derived by IP from transfected HEK-293T cells. Following kinase reactions, soluble LRRK2 and ‘on-bead’ substrates were separated and resolved on independent SDS–PAGE gels, as indicated. Western blot analysis with anti-GFP, anti-myc or anti-FLAG antibodies indicate equal loading of ArfGAP1, Dnm1, Drp1, Mfn1, Mfn2, OPA1 and LRRK2 proteins in each condition. Autoradiographs (^33^P) reveal the LRRK2-dependent phosphorylation of ArfGAP1, Dnm1, Drp1, Mfn1 and OPA1, with enhanced phosphorylation by G2019S LRRK2 compared with WT or kinase-inactive D1994A LRRK2. A soluble eluate from FLAG IPs (derived from non-transfected cells) was used as a control in each assay to assess background ^33^P incorporation for each substrate. LRRK2 autophosphorylation is also detected in these assays. Blots are representative of at least three-independent kinase experiments.

**Figure 8.**
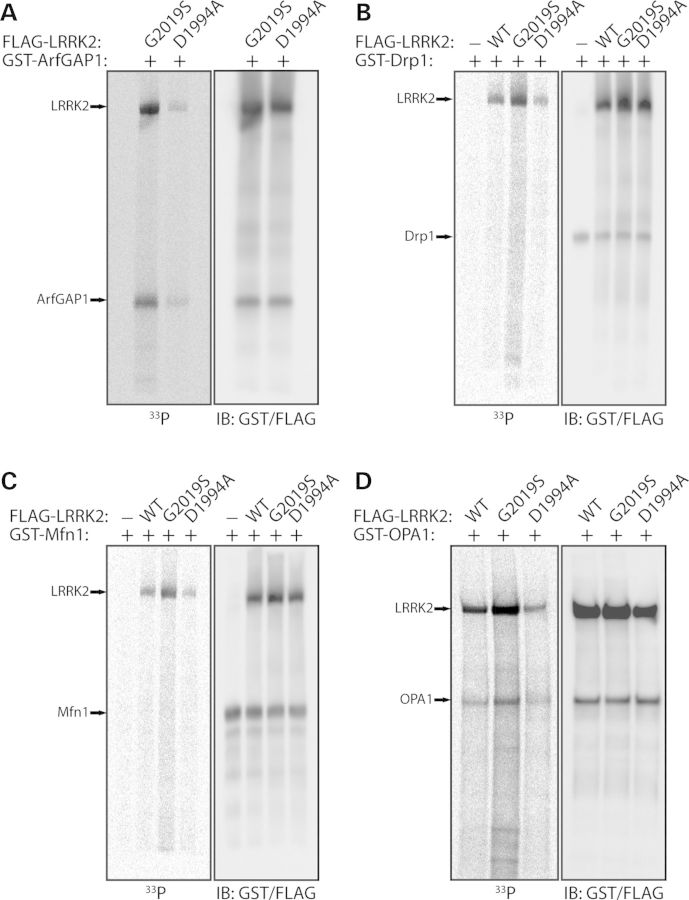
Recombinant OPA1 is phosphorylated by LRRK2 *in vitro. In vitro* kinase assays with [^33^P]-γ-ATP, recombinant soluble full-length FLAG-tagged LRRK2 variants (WT, G2019S or D1994A) and recombinant full-length GST-tagged ArfGAP1 (**A**), Drp1 (**B**), Mfn1 (**C**) or OPA1 (**D**). Western blot analyses with anti-GST and anti-FLAG antibodies indicate equal loading and positions of ArfGAP1, Drp1, Mfn1, OPA1 and LRRK2 proteins in each condition. Autoradiographs (^33^P) reveal the LRRK2-dependent phosphorylation of ArfGAP1 and OPA1, with enhanced phosphorylation by G2019S LRRK2 compared with WT or D1994A LRRK2. A soluble eluate from FLAG IPs (derived from non-transfected cells) was used as a control in each assay to assess background ^33^P incorporation for each substrate. LRRK2 autophosphorylation is also detected in these assays. Blots are representative of at least three-independent kinase experiments.

To further characterize the interaction of LRRK2 with dynamin GTPases, we sought to determine whether they serve as substrates of LRRK2-mediated phosphorylation. *In vitro* kinase assays using [^33^P]-γ-ATP with soluble recombinant full-length LRRK2 variants and each immunopurified (‘on-bead’) dynamin GTPase reveal a modest increase in the phosphorylation of Dnm1, Drp1, Mfn1 and OPA1, but not Mfn2, by G2019S LRRK2 compared with WT or kinase-inactive (D1994A) LRRK2 (Fig. 7). For comparison, we observe robust LRRK2 autophosphorylation in these kinase assays, and the robust phosphorylation of YFP-tagged ArfGAP1, a recently identified LRRK2 substrate (Fig. 7) (21,22). To reduce potential contamination from co-purified kinases in these assays, we conducted *in vitro* kinase assays using recombinant full-length GST-tagged Drp1, Mfn1 and OPA1 combined with soluble recombinant full-length LRRK2 variants in the absence of Dynabead complexes. In these assays, we consistently observe the phosphorylation of OPA1 by G2019S LRRK2, but we fail to observe appreciable phosphorylation of Drp1 and Mfn1 by LRRK2 variants despite detecting robust phosphorylation of recombinant GST-tagged ArfGAP1 by G2019S LRRK2 as well as LRRK2 autophosphorylation (Fig. 8). Our data suggest that dynamin GTPases serve as modest substrates of LRRK2-mediated phosphorylation *in vitro*, especially Dnm1 and OPA1, and should therefore be considered as putative substrates.

### Dynamin-related GTPases are not required for LRRK2-mediated toxicity in yeast

**Figure 9.**
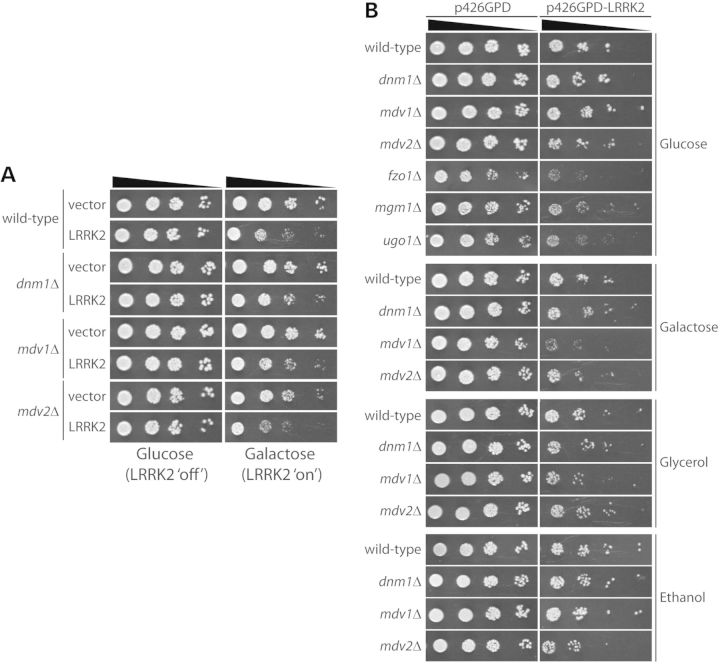
Mitochondrial dynamin GTPases are not required for LRRK2-induced toxicity in yeast. Yeast cells (BY4741 MATa), either WT or deletion mutants, were transformed with (**A**) galactose-inducible or (**B**) constitutively expressing (p426GPD) high-copy expression constructs containing human LRRK2 (residues 1300–2527). A corresponding empty vector (p426GAL1 or p426GPD) was used as a control. Cells were spotted onto SC-URA media containing glucose, galactose, glycerol or ethanol as the sole carbon source, as indicated, and incubated at 30°C for up to 6 days. Shown are 5-fold serial dilutions (from left to right) starting with equal numbers of cells. Data are representative of two independently transformed clones for each plasmid.

We previously developed a robust model of LRRK2-mediated toxicity in the baker's yeast, *Saccharomyces cerevisiae*, which exhibits defects in vesicular trafficking (14). The inducible expression of human LRRK2 fragments in yeast reduces cell viability in a manner dependent on its GTPase domain and GTPase activity (14). To further explore the functional interaction between LRRK2 and dynamin GTPases, we assessed the requirement of dynamin orthologs for LRRK2-induced toxicity in yeast. We obtained haploid yeast knockout strains harboring deletions of *DNM1*, *FZO1* and *MGM1*, which encode orthologs of mammalian Drp1, Mfn1/2 and OPA1, respectively. We also selected additional yeast knockout strains with deletion of *MDV1* (FIS2) and *MDV2* (FIS1) that are required for mitochondrial fission together with DNM1, and *UGO1* that is required for mitochondrial fusion together with FZO1 and MGM1. Yeast do not contain a clear ortholog of mammalian dynamin-1. Yeast strains were transformed with high-copy galactose-inducible (*GAL1* promoter) expression vectors containing WT human LRRK2 (residues 1300–2527) or a control empty vector, and resulting yeast clones were spotted as 5-fold serial dilutions on solid media containing glucose (LRRK2 ‘repressed’) or galactose (LRRK2 ‘induced’) to assess growth fitness. The expression of human LRRK2 impairs yeast growth on galactose media compared with a control empty vector in WT parental yeast (BY4741) (Fig. 9A). Deletion of mitochondrial fission (*DNM1*, *MDV1* and *MDV2*) factors does not influence the level of LRRK2-induced toxicity in yeast (Fig. 9A). Notably, however, *FZO1*, *MGM1* and *UGO1* knockout strains grow poorly on galactose-containing media and were refractory to further analysis.

Since yeast metabolize glucose and galactose primarily through fermentation, we conducted similar genetic interaction studies on non-fermentable carbon sources such as glycerol and ethanol that are metabolized by mitochondrial respiration (oxidative phosphorylation). We transformed yeast strains with high-copy vectors expressing human LRRK2 (residues 1300–2527) from a constitutive *GPD* promoter and conducted spotting growth assays on media containing glucose, galactose, glycerol or ethanol as the sole carbon source. *FZO1*, *MGM1* and *UGO1* deletion strains fail to grow on non-fermentable carbon sources and galactose irrespective of LRRK2 expression indicating a pronounced defect in mitochondrial respiration (Fig. 9B). However, the deletion of *FZO1*, *MGM1* and *UGO1* does not appreciably influence LRRK2-induced yeast toxicity relative to WT yeast and a control empty vector on media containing glucose (Fig. 9B). Similarly, the deletion of *DNM1*, *MDV1* and *MDV2* does not alter LRRK2-induced toxicity on media containing glucose, galactose, glycerol or ethanol as the carbon source compared with WT yeast (Fig. 9B). Of note, LRRK2-induced toxicity in WT yeast is similar on fermentable (i.e. glucose or galactose) and non-fermentable (i.e. glycerol or ethanol) carbon sources, suggesting that mitochondrial respiration is not critically required for LRRK2-mediated toxicity in yeast (Fig. 9B). Collectively, these data demonstrate that LRRK2-induced toxicity in yeast does not require endogenous dynamin-related GTPases that regulate mitochondrial fission and fusion processes, or mitochondrial respiration.

### LRRK2 attenuates neurite shortening induced by dynamin-1 by reducing its steady-state levels

**Figure 10.**
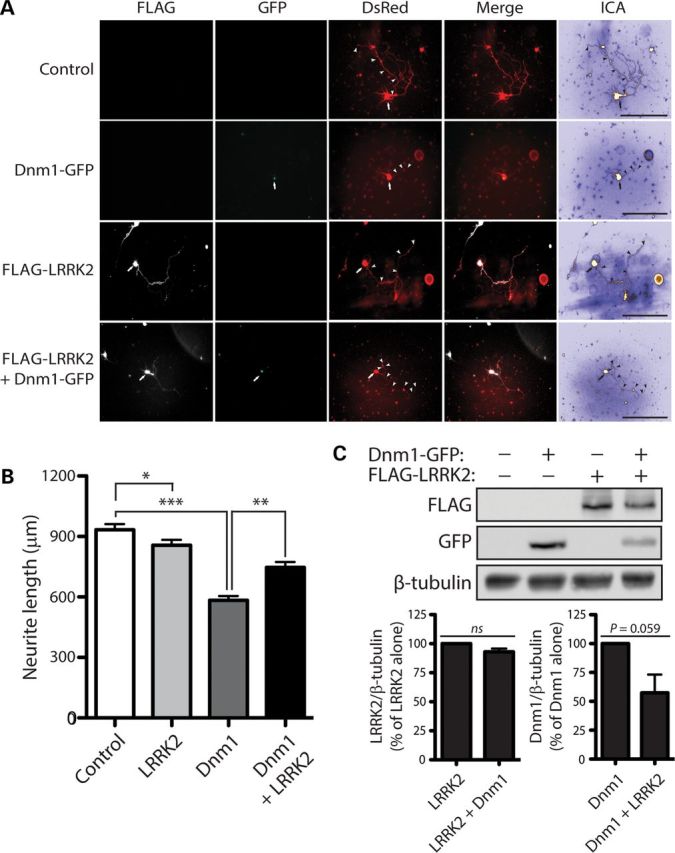
LRRK2 attenuates neurite shortening induced by Dnm1*.* (**A**) Primary cortical neurons were co-transfected with FLAG-tagged WT LRRK2, GFP-tagged Dnm1 and DsRed-Max constructs at a molar ratio of 10:10:1 at DIV 3 and fixed at DIV 7. Fluorescent microscopic images indicate the co-labeling of cortical neurons with combinations of FLAG-LRRK2, Dnm1-GFP and DsRed. DsRed images were pseudo-colored with ICA for neurite length measurements. Neuronal soma (arrows) and axonal processes (arrowheads) are indicated. Scale bars: 400 µm. (**B**) Analysis of DsRed-positive neurites reveals a marked shortening of axonal processes by Dnm1 expression alone, with a modest effect of WT LRRK2 expression alone, compared with control neurites (DsRed alone). Co-expression of WT LRRK2 and Dnm1 markedly attenuates the Dnm1-induced shortening of axonal processes. Bars represent axonal process length (mean ± SEM) expressed as a percent of DsRed alone (control) from ≥90 DsRed-positive neurons taken from at least three-independent experiments/cultures. *P < 0.05, **P < 0.01 or ***P < 0.001 by one-way ANOVA with Newman–Keuls *post hoc* analysis. (**C**) Western blot analysis with anti-FLAG, anti-GFP and anti-β-tubulin antibodies of cell extracts derived from rat primary cortical neurons at DIV 7 transiently expressing FLAG-LRRK2 and Dnm1-GFP. Densitometric analysis reveals a strong trend (*P* = 0.059 by unpaired Student's *t*-test) towards reduced Dnm1 levels in the presence of LRRK2. Graphs indicate LRRK2 (left) or Dnm1 (right) steady-state levels normalized to β-tubulin levels, expressed as a percent of each protein alone (mean ± SEM, *n* = 3 experiments). n.s., non-significant.

LRRK2 robustly regulates neuronal process complexity in primary cultures with the overexpression of PD-associated mutants leading to reduced neurite length and branching and oppositely deletion or silencing of LRRK2 enhancing neurite complexity (15,21,29,32). We therefore sought to determine the functional effects of dynamin GTPases on LRRK2-induced neurite shortening. Primary cortical neurons at days *in vitro* (DIV) 3 were co-transfected with combinations of FLAG-LRRK2, GFP-Dnm1 and DsRed-Max plasmids at a DNA molar ratio of 10:10:1 to morphologically label transfected neurons (Fig. 10A). At DIV 7, the length of DsRed-positive neuritic processes was determined for each condition. Unexpectedly, the overexpression of Dnm1 alone reduces the length of DsRed-positive neuritic processes by ∼30%, whereas expression of WT LRRK2 has modest effects on neurite length, compared with neurons expressing DsRed alone (Fig. 10B). Unexpectedly, co-expression with WT LRRK2 significantly attenuates neurite shortening induced by Dnm1 (Fig. 10B). Neurite shortening induced by the expression of K44A-Dnm1, a dominant-negative mutant that inhibits endocytosis, is also significantly attenuated by co-expression with WT LRRK2 (Supplementary Material, Fig. S3A). To potentially explain the protective effects of WT LRRK2, we assessed the steady-state levels of Dnm1-GFP and FLAG-LRRK2 proteins in cortical cultures under similar conditions. We find that LRRK2 expression results in a marked reduction of WT and K44A Dnm1 levels, whereas oppositely K44A Dnm1 but not WT Dnm1 expression markedly reduces LRRK2 levels (Fig. 10C and Supplementary Material, Fig. S3B). These data suggest that LRRK2 attenuates Dnm1-mediated neurite shortening by reducing the levels of exogenous Dnm1 protein in cortical neuronal cultures. Moreover, enhancing (WT Dnm1) or inhibiting (dominant-negative K44A Dnm1) dynamin-dependent endocytosis similarly leads to impaired neurite outgrowth.

To further understand the effect of LRRK2 on the steady-state levels of Dnm1, we assessed Dnm1 protein solubility, degradation and turnover in the presence or absence of LRRK2 in primary cortical neurons. We demonstrate that LRRK2 expression does not reduce the steady-state levels of soluble Dnm1-GFP by promoting its degradation, since prolonged inhibition of the proteasome (MG132) or lysosome (bafilomycin A1) fails to appreciably restore Dnm1-GFP levels (Supplementary Material, Fig. S4A). LRRK2 expression also fails to alter the detergent solubility of Dnm1-GFP as revealed by comparing Triton-soluble and Triton-insoluble (RIPA-soluble) neuronal extracts (Supplementary Material, Fig. S4A). Finally, we assessed the effect of LRRK2 expression on Dnm1-GFP turnover by inhibiting new protein synthesis by treatment with cycloheximide over a 24 h period in transfected neuronal cultures. Dnm1-GFP levels are relatively stable over a 24 h period with little change in turnover (Supplementary Material, Fig. S4B). LRRK2 expression fails to alter the rate of Dnm1-GFP turnover in cortical neurons (Supplementary Material, Fig. S4B). Our data suggest that the reduction in Dnm1 levels induced by LRRK2 expression in cortical neurons does not result from alterations in Dnm1 protein solubility, degradation or turnover.

### Drp1-induced neurite shortening is not influenced by LRRK2

**Figure 11.**
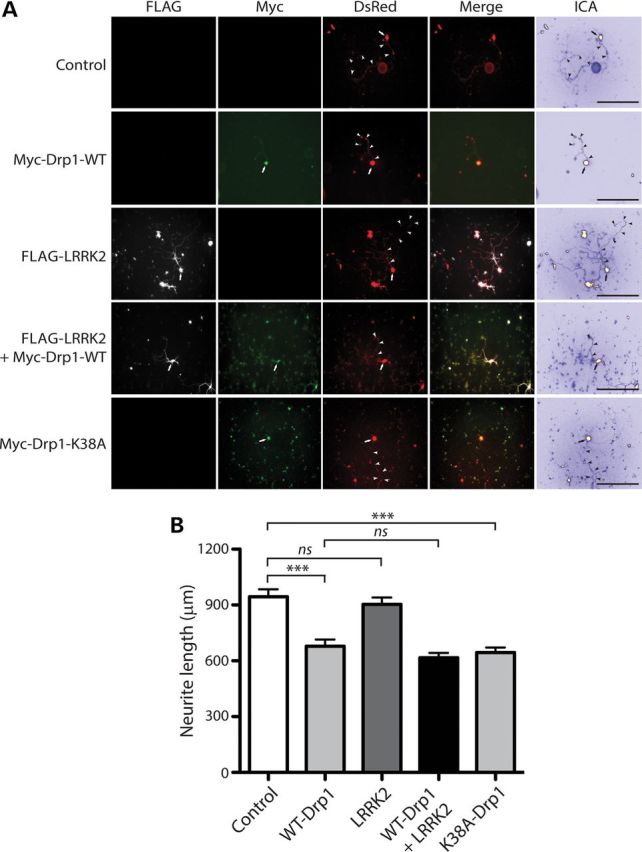
LRRK2 does not influence neurite shortening induced by Drp1*.* (**A**) Primary cortical neurons were co-transfected with FLAG-tagged WT LRRK2, Myc-tagged Drp1 (WT or K38A) and DsRed-Max constructs at a molar ratio of 10:10:1 at DIV 3 and fixed at DIV 7. Fluorescent microscopic images indicate the co-labeling of cortical neurons with combinations of FLAG-LRRK2, Myc-Drp1 and DsRed. DsRed images were pseudo-colored with ICA for neurite length measurements. Neuronal soma (arrows) and axonal processes (arrowheads) are indicated. Scale bars: 400 µm. (**B**) Analysis of DsRed-positive neurites reveals a marked shortening of axonal processes by WT or K38A Drp1 expression alone, with a negligible effect of WT LRRK2 expression alone, compared with control neurites (DsRed alone). Co-expression of WT LRRK2 and WT Drp1 fails to alter Drp1-induced shortening of axonal processes. Bars represent axonal process length (mean ± SEM) expressed as a percent of DsRed alone (control) from ≥90 DsRed-positive neurons taken from at least three-independent experiments/cultures. ***P < 0.001 by one-way ANOVA with Newman–Keuls *post hoc* analysis. n.s., non-significant.

Next, the effects of Drp1 on LRRK2-induced neurite shortening were assessed. Primary cortical neurons were co-transfected with combinations of FLAG-LRRK2, Myc-Drp1 and DsRed-Max plasmids and the length of DsRed-positive neuritic processes were determined (Fig. 11A). The overexpression of WT or K38A Drp1 alone significantly reduces neurite length by ∼30% with negligible effects of WT LRRK2 alone, whereas co-expression with WT LRRK2 fails to influence the effects of WT Drp1 on neurite length (Fig. 11B). Although Myc-Drp1 protein is detectable by immunofluorescence analysis in these cultures (Fig. 11A), it was not possible to reliably assess the levels of Myc-Drp1 protein by western blot analysis under similar conditions due to the low expression of exogenous Drp1 (data not shown). Our data indicate that LRRK2 does not influence Drp1-mediated neurite shortening potentially suggesting that Drp1 lies downstream of LRRK2 activity in a common pathway. Moreover, enhancing (WT Drp1) or inhibiting (dominant-negative K38A Drp1) mitochondrial fission similarly leads to impaired neurite outgrowth (Fig. 11B).

### Neurite shortening induced by mitofusin-1 is attenuated by LRRK2

**Figure 12.**
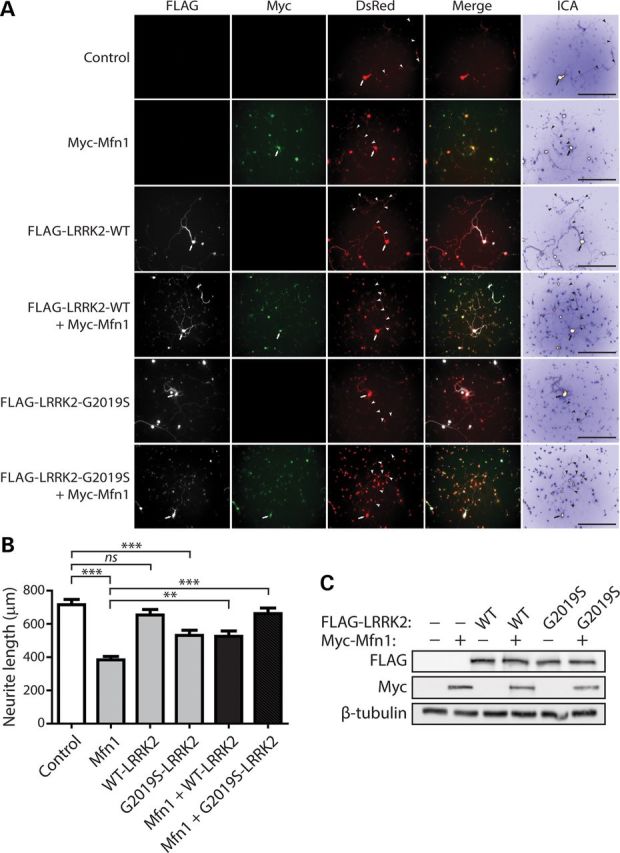
LRRK2 rescues impaired neurite outgrowth induced by Mfn1*.* (**A**) Primary cortical neurons were co-transfected with FLAG-tagged LRRK2 (WT or G2019S), Myc-tagged Mfn1 and DsRed-Max constructs at a molar ratio of 10:10:1 at DIV 3 and fixed at DIV 7. Fluorescent microscopic images indicate the co-labeling of cortical neurons with combinations of FLAG-LRRK2, Myc-Mfn1 and DsRed. DsRed images were pseudo-colored with ICA for neurite length measurements. Neuronal soma (arrows) and axonal processes (arrowheads) are indicated. Scale bars: 400 µm. (**B**) Analysis of DsRed-positive neurites reveals a marked shortening of axonal processes by Mfn1 or G2019S LRRK2 expression alone, with a negligible effect of WT LRRK2 expression alone, compared with control neurites (DsRed alone). Co-expression of G2019S LRRK2 and Mfn1 rescues the Mfn1-induced shortening of axonal processes, whereas WT LRRK2 partially rescues the effects of Mfn1. Bars represent axonal process length (mean ± SEM) expressed as a percent of DsRed alone (control) from ≥90 DsRed-positive neurons taken from at least three-independent experiments/cultures. **P < 0.01 or ***P < 0.001 by one-way ANOVA with Newman–Keuls *post hoc* analysis. n.s., non-significant. (**C**) Western blot analysis with anti-FLAG, anti-myc and anti-β-tubulin antibodies of cell extracts derived from rat primary cortical neurons at DIV 7 transiently expressing FLAG-LRRK2 and Myc-Mfn1. The levels of LRRK2 or Mfn1 are not altered when expressed alone or together. Blots are representative of three-independent experiments.

To explore the functional effects of Mfn1 on LRRK2-induced neurite shortening, we conducted similar assays in primary cortical neurons. Similar to Dnm1 and Drp1, the overexpression of Mfn1 alone dramatically reduces the length of DsRed-positive neuritic processes by >40% compared with the expression of WT LRRK2 or DsRed alone (Fig. 12A and B). Co-expression with WT LRRK2 partially rescues Mfn1-induced neurite shortening (Fig. 12A and B). We conducted similar experiments using G2019S LRRK2. The overexpression of G2019S LRRK2 alone causes a ∼30% reduction in neurite length, whereas surprisingly, its co-expression with Mfn1 leads to a complete rescue of Mfn1-induced neurite shortening (Fig. 12A and B). Under similar conditions, the steady-state levels of exogenous FLAG-LRRK2 and Myc-Mfn1 proteins in cortical neurons assessed by western blot analysis are similar when expressed alone or together, indicating that the effects of these proteins on neurite length do not result from altered protein levels (Fig. 12C). Our data indicate that LRRK2 rescues Mfn1-mediated neurite shortening potentially by promoting mitochondrial fission and thereby counteracting excessive mitochondrial fusion induced by Mfn1 expression. Moreover, enhancing mitochondrial fusion (WT Mfn1, Fig. 12) or inhibiting mitochondrial fission (K38A Drp1, Fig. 11) similarly result in impaired neurite outgrowth.

## DISCUSSION

Here, we identify and functionally validate novel interactions of LRRK2 with multiple members of the dynamin GTPase superfamily that are known to regulate membrane dynamics (52). LRRK2 commonly interacts with the classical dynamins, Dnm1, Dnm2 and Dnm3, which play an important role in membrane scission during clathrin-mediated endocytosis. LRRK2 also interacts with dynamin-related GTPases that regulate mitochondrial membrane fission (Drp1) and fusion (Mfn1, Mfn2 and OPA1) events. The full-length LRRK2 protein is required for the interaction with OPA1, whereas multiple N-terminal (LRRK2-specific, armadillo, ankyrin, LRR and Roc domains) and C-terminal (COR and WD40 domains) domains of LRRK2 are sufficient for the interaction with Dnm1 or Mfn1. PD-associated mutations located within the Roc (R1441C), COR (Y1699C) and kinase (G2019S) domains of LRRK2 do not influence the interaction with Dnm1, Mfn1 or OPA1. LRRK2 partially co-localizes with Dnm1 upon early endosomal membranes, with Drp1 mostly in the cytoplasm, and with Mfn1, Mfn2 and OPA1 at mitochondrial membranes of neural cells. Furthermore, dynamin GTPases co-fractionate with endogenous LRRK2 in synaptosomes and microsomes from mouse brain, although the expression of human G2019S LRRK2 does not influence their subcellular distribution. Dynamin GTPases form native oligomeric complexes in mouse brain that are not altered by modulating LRRK2 expression. Furthermore, the steady-state levels of S-OPA1 are reduced in G2019S mutant PD brains, whereas other dynamin GTPases are not altered by the G2019S mutation. Dynamin-related GTPases but not Dnm1 tend to promote the GTP-binding capacity of LRRK2, whereas oppositely LRRK2 promotes the binding of Mfn1 to GTP. Dynamin GTPases serve as modest substrates of LRRK2-mediated phosphorylation *in vitro*, especially Dnm1 and OPA1. In a yeast model, endogenous dynamin GTPases that regulate mitochondrial fission and fusion, as well as mitochondrial respiration, are not required for LRRK2-mediated cellular toxicity. Alternatively, however, LRRK2 functionally interacts with dynamin GTPases in the regulation of neurite outgrowth in cultured primary cortical neurons. LRRK2 attenuates impaired neurite outgrowth induced by Dnm1 through a reduction in the levels of Dnm1 protein. Drp1 and LRRK2 do not appear to functionally interact upon neurite complexity, whereas LRRK2 can rescue impaired neurite outgrowth induced by Mfn1. Collectively, our data support the novel biochemical and selective functional interaction of LRRK2 with members of the dynamin GTPase superfamily that implicate LRRK2 in the regulation of membrane dynamics involved in endocytosis and mitochondrial function.

Our studies have previously implicated LRRK2 in regulating endocytosis and exocytosis (14). The overexpression of human LRRK2 in cultured neurons delayed synaptic vesicle endocytosis and exocytosis, whereas LRRK2 expression in yeast cells disrupted endocytic trafficking to the vacuole coincident with the accumulation of autophagic vacuoles (14). A recent study has shown that LRRK2 may control synaptic vesicle storage and mobilization within the recycling pool through an unclear mechanism although LRRK2 was found to putatively interact with a number of proteins in synaptosomes involved in vesicular recycling including Dnm1 (24). Here, we describe a robust biochemical interaction between LRRK2 and Dnm1 that is not influenced by PD-associated mutations and most likely occurs at the cytoplasmic face of early endosomal vesicles. LRRK2 and Dnm1 do not appear to influence the GTPase activity of each other insofar as GTP-binding capacity is unaffected, whereas Dnm1 serves as a modest substrate of LRRK2-mediated phosphorylation *in vitro*. Instead, LRRK2 and Dnm1 functionally interact upon neurite complexity where gain (WT) or loss (K44A) of Dnm1 function impairs neurite outgrowth that can be rescued by expression of WT LRRK2 by reducing exogenous Dnm1 levels. The reduction of Dnm1 by WT LRRK2 could potentially result from increased protein degradation or impaired transcription/translation, or could reflect movement of Dnm1 into a biochemically insoluble cellular compartment. However, we did not observe any impact of G2019S LRRK2 expression on the subcellular fractionation profile or steady-state levels of endogenous Dnm1 in mouse and human brain nor could we observe reduced Dnm1 levels by LRRK2 in HEK-293T cells using exogenous proteins (refer to Figs 1A and G, 4C and 5). We further explored the fate of exogenous Dnm1 in primary cortical neurons and could demonstrate that WT LRRK2 expression did not alter the detergent solubility of Dnm1 whereas proteasome or lysosome inhibition failed to recover Dnm1 protein levels. Furthermore, protein turnover assays with cycloheximide in cortical neurons did not reveal altered stability of Dnm1 in the presence of LRRK2. Collectively, these observations suggest that LRRK2 may influence the transcription and/or translation of exogenous Dnm1, an effect that may be specific to cortical neurons and/or exogenously expressed (rather than endogenous) Dnm1. Taken together, the functional significance of the robust biochemical interaction between LRRK2 and Dnm1 is unclear at present, although it is possible that LRRK2 exists in a protein complex with Dnm1 and exerts subtle effects on its function. Our findings further support a connection of LRRK2 with the regulation of endocytosis and membrane dynamics potentially through Dnm1.

We have previously shown that a small proportion of endogenous LRRK2 is associated with mitochondria by submitochondrial fractionation and electron microscopic analysis of rodent brain (39). More recent studies have shown that LRRK2 can regulate mitochondrial activity and morphology in various cellular models and mutant LRRK2 transgenic mice, although through an unclear mechanism (27,32,36–38). Mutant LRRK2 has recently been shown to induce mitochondrial fission in a Drp1-dependent manner, potentially through their direct interaction, and restoring mitochondrial morphology by inhibiting fission attenuated LRRK2-mediated neuronal toxicity (49,50). These initial observations suggest that mitochondrial fission is required for LRRK2-induced neuronal damage. Here, we further extend these previous studies by demonstrating that LRRK2 commonly and robustly interacts with multiple dynamin-related GTPases that regulate mitochondrial fission and fusion, including Drp1, Mfn1, Mfn2 and OPA1. Dynamin-related GTPases tend to enhance the GTP-binding capacity of LRRK2, whereas LRRK2 reciprocally increases the level of GTP-bound Mfn1. Increased GTP-binding capacity could reflect a GTP-bound ‘active’ state of LRRK2 and Mfn1, or alternatively may suggest an impairment of GTP hydrolysis. GTPase activity is critical for the membrane fusion activity of Mfn1 and for the proper function of LRRK2 (23,54). Recombinant OPA1 (but not Drp1 or Mfn1) could serve as a modest substrate of LRRK2-mediated phosphorylation *in vitro* but the impact of phosphorylation on OPA1 function and whether and to what degree this occurs in mammalian cells or tissues remains to be clarified. Despite the effects of LRRK2 on the GTP-binding capacity and phosphorylation of dynamin-related GTPases, the subcellular distribution, steady-state levels and native oligomeric complexes of dynamin GTPases were not altered by G2019S LRRK2 expression or LRRK2 deletion in the mammalian brain. While human LRRK2-induced toxicity in yeast does not require endogenous dynamin-related GTPase orthologs (i.e. DNM1/Drp1, FZO1/Mfn or MGM1/OPA1) or mitochondrial respiration, LRRK2 instead functionally interacts with Mfn1 but not Drp1 upon neurite complexity, with WT and G2019S LRRK2 rescuing impaired neurite outgrowth induced by Mfn1 overexpression. These data suggest that excessive mitochondrial fusion through Mfn1 overexpression promotes neuronal toxicity, and that potentially the profission effects of LRRK2 (with G2019S>WT) can reverse and mitigate these neurite effects. It is also plausible that LRRK2 could directly interact with and inactivate Mfn1 and thus inhibit its profusion effects, as reflected by the increased levels of GTP-bound Mfn1 induced by LRRK2 that may reflect impaired GTPase activity. We also observe weak phosphorylation of immunopurified Mfn1 by G2019S LRRK2 *in vitro* and whether this occurs *in vivo* and contributes to Mfn1 function is not yet clear. The lack of functional interaction in yeast between human LRRK2 and FZO1, an ortholog of mammalian Mfn1/2, could suggest that FZO1 and Mfn1 are not entirely functionally conserved or that the functional interaction of LRRK2 and Mfn1 is context dependent and restricted to mammalian cells or neurons. It is interesting to note that mitochondrial fragmentation and neuronal cell death induced by mutant LRRK2 could be attenuated by inhibiting mitochondrial fission via overexpression of dominant-negative Drp1 (K38A) (50), suggesting that increasing mitochondrial fusion in the context of mutant LRRK2 is beneficial. Our data would suggest that excessive mitochondrial fission (via WT Drp1) or fusion (via WT Mfn1 or K38A Drp1) are in general both detrimental in the context of neurite outgrowth. The lack of an apparent functional interaction between LRRK2 and Drp1 in these neurite outgrowth assays could suggest that LRRK2 operates upstream of Drp1 in a common pathway, where mitochondrial fission and impaired neurite outgrowth induced by Drp1 overexpression potentially supersedes the requirement for LRRK2-mediated activation of Drp1. This would be consistent with prior observations that LRRK2 induces mitochondrial fission and neuronal cell death in a Drp1-dependent manner (49,50).

Collectively, our data support the broad and robust interaction of LRRK2 with members of the dynamin GTPase superfamily in mammalian cells. LRRK2 appears to exhibit distinct functional effects upon individual dynamin GTPases. In particular, LRRK2 promotes the GTP-binding capacity of Mfn1 and rescues impaired neurite outgrowth induced by Mfn1. Alternatively, LRRK2 modestly phosphorylates OPA1 *in vitro* and the levels of mature S-OPA1 are reduced in G2019S PD brains. Therefore, the functional interaction of LRRK2 with dynamin GTPases is rather complex but provides initial mechanistic support of a role for LRRK2 in regulating membrane dynamics important for mediating endocytosis and mitochondrial function (14,24–27,32,36–38). Future studies are warranted to clarify the physiological or pathological interaction of LRRK2 with dynamin GTPases in animal models. Our findings suggest that dynamin GTPases represent an important family of proteins implicated in the biology and pathophysiology of LRRK2.

## MATERIALS AND METHODS

### Ethics statement

For use of human brain tissue in this study, patients provided written informed consent and approval for the consent procedure and experiments were obtained from the NHS National Research Ethics Committee of the UK (Approval No. 02/N093). All animal experiments were approved by the SCAV (Service de la consommation et des affaires veterinaires) in the Canton de Vaud, Switzerland (Animal authorization No. 2293), and conducted in strict accordance with the European Union directive (2010/63/EU) for the care and use of laboratory animals.

### Animals

Mice and rats were maintained in a pathogen-free barrier facility and exposed to a 12 h light/dark cycle with food and water provided *ad libitum*. Pregnant female Sprague-Dawley rats were obtained from Charles River Laboratories (L'Arbresle Cedex, France) and resulting P1 rats were used for preparation of primary cortical neuronal cultures. LRRK2 knockout mice with a deletion of exon 41 (57) were kindly provided by Drs. Giorgio Rovelli and Derya Shimshek (Novartis Pharma AG, Basel, Switzerland). Transgenic mice expressing full-length human G2019S LRRK2 from a CMV-enhanced PDGFβ promoter (line 340) were described previously (32).

### Expression plasmids, proteins and antibodies

Mammalian expression plasmids containing codon-optimized FLAG-tagged full-length human LRRK2 (WT, R1441C, Y1699C and G2019S) and LRRK2 deletion mutants were kindly provided by Dr. Christopher Ross (Johns Hopkins University, Baltimore, MD, USA). T1348N and D1994A mutations were introduced into FLAG-tagged WT LRRK2 as previously described (21). GFP-tagged human dynamin-1 (WT and K44A) plasmids were kindly provided by Dr. Pietro De Camilli (Yale University, New Haven, CT, USA) (58), and YFP-tagged ArfGAP1 was provided by Dr. Jennifer Lippincott-Schwartz (National Institutes of Health, Bethesda, MD, USA) (59). As plasmid controls, pcDNA3.1 (Invitrogen) and pDsRed-Max-N1 (Addgene #21718) plasmids were obtained. HA-tagged mouse Dnm1 (#36263), Dnm2 (#36264) and Dnm3 (#36265) and HA-tagged human Dnm1 (#34682) plasmids were from Addgene. Myc-tagged human Drp1 (WT and K38A), Mfn2 and OPA1, and mito-RFP plasmids were kindly provided by Dr. Manuel Rojo (Université Victor Segalen, France). 10xMyc-tagged mouse Mfn1 (WT, #23212 and K88T, #26050) plasmids were obtained from Addgene. Expression plasmids containing human RFP-Rab5A (#14437) and human GFP-Rab7A (#12605) were obtained from Addgene. GST-tagged full-length human ArfGAP1 (residues 1–415), Drp1 (residues 1–711), Mfn1 (residues 1–742) and OPA1 (residues 1–960) proteins were obtained from Novus Biologicals (Littleton, CO, USA). The following antibodies were employed: mouse monoclonal anti-FLAG-(M2), anti-FLAG-(M2)-peroxidase, anti-β-tubulin (clone TUB 2.1), and rabbit polyclonal anti-βIII-tubulin and anti-Mitofusin 2 (Sigma-Aldrich); mouse monoclonal anti-GFP (clones 7.1 and 13.1), anti-c-myc (clone 9E10) and anti-c-myc-peroxidase, and rat monoclonal anti-HA (clone 3F10) (Roche Applied Science); rabbit monoclonal anti-LRRK2 (clone MJFF4/c81-8; Epitomics Inc.); mouse monoclonal anti-LRRK2 (clone N241A/34; UC Davis/NIH NeuroMab); mouse monoclonal anti-synaptophysin 1 (Synaptic Systems); rabbit polyclonal anti-Dynamin-1 (ThermoFisher Scientific); mouse monoclonal anti-OPA1 (clone 18/OPA-1) and anti-DLP1/Drp1 (clone 8/DLP1) (BD Biosciences); rabbit polyclonal anti-GST and anti-myc (Covance); peroxidase-coupled anti-mouse, anti-rabbit and anti-rat IgG, light chain-specific secondary antibodies (Jackson ImmunoResearch Inc.); anti-rabbit, anti-mouse and anti-rat IgG coupled to AlexaFluor-488, -546 and -633 (Invitrogen).

### Cell culture and transient transfection

HEK-293T and SH-SY5Y neuroblastoma cells were maintained in Dulbecco's modified Eagle's media supplemented with 10% fetal bovine serum and 1× penicillin/streptomycin at 37°C in a humidified atmosphere containing 5% CO_2_. Cells were transfected with plasmid DNAs using X-tremeGENE HP DNA Transfection Reagent (Roche Applied Science). Cells were routinely harvested at 48–72 h posttransfection for biochemical assays.

### Co-immunoprecipitation assays and western blotting

Co-IP assays were conducted as previously described (21). Briefly, HEK-293T cells were harvested in IP buffer (1× PBS, pH 7.4, 1% Triton X-100, 1× phosphatase inhibitor cocktail 2 and 3 [Sigma-Aldrich], 1× Complete Mini Protease Inhibitor cocktail [Roche Applied Sciences]) and incubated overnight at 4°C with Protein G-Dynabeads (Invitrogen) pre-coupled with mouse anti-FLAG-M2 (5 µg; Sigma-Aldrich), rat anti-HA (2 µg; Roche Applied Science), mouse anti-myc (5 µg; Roche Applied Science) or mouse anti-GFP (1 µg; Roche Applied Science) antibodies. Dynabead complexes were washed with IP buffer and proteins eluted at 70°C for 10 min in Laemmli sample buffer (Bio-Rad) containing 5% 2-mercaptoethanol. IPs and input lysates (1% total) were resolved by SDS–PAGE, transferred to Protran nitrocellulose (0.2 µm; Perkin Elmer), and subjected to western blotting with appropriate primary and secondary antibodies. Proteins were visualized by enhanced chemiluminescence (ECL; GE Healthcare) on a FujiFilm LAS-4000 Luminescent Image Analysis system. LabImage 1D software (Kapelan Bio-Imaging Solutions) was used for quantitation of protein levels by densitometric analysis.

For *in vivo* co-IP, protein extracts were prepared from whole brains of adult WT and LRRK2 knockout mice (with targeted deletion of exon 41 of the *LRRK2* gene (57); kindly provided by Drs. Giorgio Rovelli and Derya Shimshek, Novartis Pharma AG, Basel, Switzerland) by homogenization in TNE buffer (10 mm Tris–HCl, pH 7.4, 150 mm NaCl, 5 mm EDTA, 0.5% NP-40, phosphatase inhibitor cocktails 2 and 3 [Sigma-Aldrich], Complete Mini protease inhibitor cocktail [Roche Applied Sciences]). Protein concentration was determined by BCA assay (Pierce Biotechnology, Rockford, IL, USA). Brain extracts (20 mg protein) were combined with 50 µl Protein G-Dynabeads (Invitrogen) pre-incubated with mouse monoclonal anti-LRRK2 (5 µg; N241A/34; NeuroMab) antibody followed by overnight incubation at 4°C. Dynabead complexes were sequentially washed twice with TNE buffer and twice with TBS buffer (10 mm Tris–HCl, pH 7.4, 150 mm NaCl). Immunoprecipitates were eluted by heating at 70°C for 10 min, resolved by SDS–PAGE and subjected to western blot analysis.

### Immunocytochemistry and confocal microscopy

SH-SY5Y cells and rat primary cortical neurons were processed for immunocytochemistry as previously described (21). Briefly, transfected cells were fixed in 4% paraformaldehyde (PFA) and incubated with combinations of mouse anti-FLAG, rat anti-HA or rabbit anti-myc antibodies together with anti-mouse IgG-AlexaFluor-546 or -633, anti-rabbit IgG-AlexaFluor-488 or -633 or anti-rat IgG-AlexaFluor-488 antibodies and stained with DAPI. Images were acquired using a Zeiss LSM 700 inverted confocal microscope (Carl Zeiss AG) with a Plan-Apochromat ×63/1.40 oil objective in *x*, *y* and *z* planes. Images were subjected to deconvolution using HuygensPro software (Scientific Volume Imaging). Representative images are taken from a single *z*-plane at 0.1 µm thickness. To provide a relative assessment of co-localization, images were minimized for background noise at acquisition and subjected to deconvolution. Images were analyzed using a co-localization threshold plugin in NIH ImageJ to calculate the intensity of pixels that overlap between the two fluorescent channels. ImageJ automatically sets a threshold for each channel and renders a gray scale 8-bit image containing only those pixels above the threshold level. A scatterplot of pixel intensity for the two channels is plotted on *x*- and *y*-axes with a linear regression fit, from which Pearson's correlation coefficient (Rcoloc) values are calculated for the two channels ranging from 0 (random co-localization) to 1 (perfect co-localization). A value of −1 indicates a perfect exclusion of the two signals.

### Size-exclusion chromatography

Size-exclusion chromatography was conducted as described previously using an Akta-FPLC system (Amersham Biosciences) (44). Briefly, cleared brain extracts prepared in lysis buffer (0.1% Triton X-100, 1× PBS, pH 7.4, 1× Complete Protease Inhibitor cocktail [Roche Applied Science]) derived from whole brains of adult WT and LRRK2 KO mice were injected for FPLC on a Superdex 200 10/300 GL column (Amersham Biosciences). The elution volumes of standards were 9 ml for thyroglobulin (669 kDa), 10.5 ml for ferritin (440 kDa), 12.5 ml for aldolase (158 kDa) and 15.5 ml for conalbumin (75 kDa). Fractions (0.5 ml) were analyzed by SDS–PAGE and western blotting with anti-Dnm1, anti-Drp1, anti-β-tubulin and anti-LRRK2 (N241A/34) antibodies.

### Native PAGE

Brain extracts were prepared from cerebral cortex tissue pooled from two adult WT and LRRK2 KO mice, or human G2019S LRRK2 transgenic and non-transgenic mice by homogenization in TEVP buffer (10 mm Tris–HCl, pH 7.4, 5 mm NaF, 1 mm Na_3_VO_4_, 1 mm EDTA, 1 mm EGTA) containing 320 mm sucrose. Equivalent extracts were resolved on Native-PAGE 3–12% Bis–Tris gradient gels (Invitrogen) and blots were subjected to western blotting with anti-Dnm1, anti-Drp1, anti-Mfn2, anti-OPA1 and anti-LRRK2 antibodies. In parallel, equivalent extracts were analyzed by SDS–PAGE and western blotting.

### Subcellular fractionation of mouse brain

Subcellular fractionation was conducted by differential centrifugation as described previously (21,44) using cerebral cortex tissue pooled from two adult WT and LRRK2 KO mice, or human G2019S LRRK2 transgenic and non-transgenic mice. Fractions generated include: total homogenate (H), nuclear/whole cell (P1), soluble cytosolic (S1, S2 and S3), heavy membrane (P2), light membrane/microsomes (P3), synaptosomal membrane (LP1) and cytosolic (LS1), synaptic vesicle-enriched (LP2) and cytosolic (LS2) fractions. Equal quantities of each fraction as determined by BCA assay (Pierce Biotechnology) were assessed by western blotting with antibodies labeling mitochondria (Mfn2 and OPA1; P2 and LP1) and synaptosomes/synaptic vesicles (synaptophysin 1; P2, P3, LP1 and LP2) subcellular compartments.

### Human brain tissue and fractionation

Human tissue for these studies was obtained from the archive at Queen Square Brain Bank (QSBB) as previously reported (44,60). Flash-frozen frontal cortex tissue derived from four G2019S PD, four idiopathic PD and four control subjects was employed. Table 1 lists the details of these human subjects. Tissue homogenates (10%, w/v) were prepared in homogenization buffer (20 mm Tris–HCl, pH 7.4, 150 mm NaCl, 1× Complete Protease Inhibitor cocktail [Roche Applied Science], 1× phosphatase inhibitor cocktail [Roche Applied Science]) as previously described (44,60). Equivalent proteins were resolved on 3–8% Tris–acetate SDS–PAGE gradient gels (Invitrogen), and blots were subjected to western blotting with anti-Dnm1, anti-Drp1, anti-Mfn2, anti-OPA1 and anti-β-tubulin antibodies.

**Table 1.** Clinical details of human brain tissue

Subject	Gender	Age (years)	PMD (h)	Pathology
G2019S 1	F	80	44.4	Limbic
G2019S 2	F	81	15	Limbic
G2019S 3	F	84	32.2	Limbic
G2019S 4	F	72	24.55	Limbic
iPD 1	F	69	52.5	Limbic
iPD 2	M	70	61.2	Limbic
iPD 3	F	87	47.45	Limbic
iPD 4	M	75	48	Limbic
Control 1	F	85	37	N/A
Control 2	M	93	112	N/A
Control 3	F	91	98.5	N/A
Control 4	M	87	36	N/A

iPD, idiopathic Parkinson's disease; Limbic, limbic subtype of Lewy body pathology according to McKeith consensus criteria for the classification of DLB** (61); N/A, non-applicable; PMD, postmortem delay.

### GTP-binding assay

HEK-293T cell extracts were prepared in buffer A (1× PBS, pH 7.4, 1% Triton X-100, 1× phosphatase inhibitor cocktail 2 and 3 [Sigma-Aldrich], 1× Complete Mini Protease Inhibitor Cocktail [Roche Applied Science]). Equivalent extracts were incubated with 25 µl of guanosine 5′-triphosphate-agarose (Sigma-Aldrich) by rotating for 2 h at 4°C. Agarose beads were washed three times with buffer A and once with 1× PBS, and GTP-bound proteins were eluted in Laemmli sample buffer containing 5% 2-mercaptoethanol and heating at 70°C for 10 min. GTP-bound fractions and input lysates (1% total) were resolved by SDS–PAGE and subjected to western blotting with anti-FLAG, anti-GFP and anti-myc antibodies.

### *In vitro* radioactive kinase assays

Kinase assays were conducted essentially as previously described (20,21). HEK-293T cell extracts expressing full-length Myc-Drp1, Myc-Mfn1, Myc-Mfn2, Myc-OPA1, Dnm1-GFP or ArfGAP1-YFP were subjected to IP with Protein G-Dynabeads (Invitrogen) precoupled to anti-myc (5 µg) or anti-GFP (1 µg) antibodies and extensive washing for ‘on-bead’ kinase assays. Soluble recombinant full-length FLAG-LRRK2 proteins were purified from cell extracts by IP with anti-FLAG-(M2)-agarose (Sigma-Aldrich) and eluted with 3xFLAG peptide. In some assays, recombinant full-length GST-tagged Drp1, Mfn1, OPA1 or ArfGAP1 proteins (1 µg/reaction; Novus Biologicals) were used together with soluble FLAG-LRRK2 proteins. A fraction of each soluble or on-bead protein preparation was routinely resolved by SDS–PAGE and stained with Coomassie G-250 (Bio-Rad) to confirm protein purity. Kinase reactions were performed with equivalent quantities of LRRK2 or substrate proteins in kinase buffer (25 mm Tris–HCl, pH 7.5, 5 mm β-glycerophosphate, 2 mm dithiothreitol, 0.1 mm Na_3_VO_4_, 10 mm MgCl_2_) in the presence of [^33^P]-γ-ATP (2 µCi/reaction; Perkin Elmer) and 5 µm cold ATP (Sigma-Aldrich) at 30°C for 1 h in a final volume of 25 µl. Reactions were terminated with 4× Laemmli buffer and heating at 95°C for 10 min. Prior to termination, kinase reactions containing soluble LRRK2 and ‘on-bead’ substrate proteins were separated. Reactions samples were resolved on 3–8% Tris–acetate or 4–16% Tris–glycine SDS–PAGE gradient gels (Invitrogen) and transferred to PVDF membranes. Incorporated radioactivity was detected using a Phosphor imaging system and membranes were subsequently probed with anti-FLAG, anti-myc, anti-GFP or anti-GST antibodies to confirm equal protein loading of LRRK2 and each substrate. Relative substrate phosphorylation levels were determined by densitometric analysis of ^33^P signals normalized to substrate protein levels on western blots.

### Yeast growth assays

A WT human LRRK2 cDNA fragment (residues 1300–2527) was amplified from a pYES2/CT-LRRK2 vector (14) by PCR to incorporate a 5′ optimal yeast Kozak sequence (AAAAATGTCT) and a 3′ in-frame C-terminal V5 tag and stop codons. Blunt-end PCR products were first cloned into a directional pENTR/D-TOPO entry vector, sequenced to confirm their integrity and subjected to recombination with the Gateway-compatible destination vectors, p426GAL1-ccdB or p426GPD-ccdB (2 µm ori, *URA3*; Addgene), for high-copy expression in yeast cells. Yeast haploid parental strain BY4741 (*MATa*, *his3Δ1*, *leu2Δ0*, *met15Δ0* and *ura3Δ0*) and deletion strains (*Δdnm1*, *Δmdv1*, *Δmdv2*, *Δfzo1*, *Δmgm1* and *Δugo1*) on a BY4741 genetic background were obtained from Open Biosystems (ThermoFisher Scientific). Yeast manipulations were performed, and media were prepared using standard procedures. Yeast cells were transformed with plasmids using a standard lithium acetate procedure. Yeast cells carrying high-copy (2 µm) expression plasmids (p426GAL1-LRRK2, p426GPD-LRRK2, p426GAL1 or p426GPD) were routinely grown in synthetic complete media lacking uracil (SC-URA) containing glucose (2% dextrose). Yeast cells were diluted in dH_2_O and normalized for OD_600_ nm, serially diluted (5-fold) and spotted onto plates containing solid media (SC-URA) with 2% glucose, galactose, glycerol or ethanol as the sole carbon source. Cells were grown at 30°C for up to 6 days before imaging.

### Primary neuronal cultures and neurite length assays

Primary cortical neurons were prepared from Sprague-Dawley P0–P1 rats by stereoscopically isolating the cerebral cortices and dissociation by digestion in media containing papain (20 U/ml; Sigma-Aldrich) and mechanical trituration. Cells were plated in 35 mm dishes on glass cover slips coated with poly-d-lysine (20 ng/ml; BD Biosciences) and mouse laminin (33 µg/ml; Invitrogen) and maintained in Neurobasal media containing B27 supplement (2%, w/v), l-glutamine (500 µm) and penicillin/streptomycin (100 U/ml) (Invitrogen).

Neurons were transfected at DIV 3 with combinations of FLAG-LRRK2, Myc-Drp1/Myc-Mfn1/Dnm1-GFP and DsRed-Max plasmids at a 10:10:1 DNA molar ratio (5 µg DNA total/dish) using Lipofectamine 2000 reagent (Invitrogen). Cultures were fixed at DIV 7 with 4% PFA and subjected to immunocytochemistry with mouse anti-FLAG antibody (Sigma-Aldrich) and rabbit anti-myc antibody (Covance), and anti-mouse IgG-AlexaFluor-633 and anti-rabbit IgG-AlexaFluor-488 antibodies (Invitrogen). Fluorescent images were acquired using an EVOS inverted fluorescent digital microscope (Advanced Microscopy Group) with a ×10 objective. DsRed images were pseudo-colored with ICA1 using NIH ImageJ software to improve the contrast of neuritic processes, and used for neurite length measurements. The length of DsRed-positive axonal processes from single-labeled (DsRed alone), double-labeled (DsRed/FLAG, DsRed/Myc or DsRed/GFP) or triple-labeled (DsRed/FLAG/Myc or DsRed/FLAG/GFP) cortical neurons were measured using the line tool function of ImageJ by an investigator blinded to each condition. Only neurons that had extended neurites were measured, whereas neurons without processes were excluded from the analysis. For each experiment, axonal processes from ≥30 DsRed-positive neurons randomly sampled across five cover slips were measured, and repeated for at least three-independent experiments/cultures.

### Statistical analyses

Data were analyzed by one-way ANOVA with Newman–Keuls *post hoc* analysis for comparison of multiple data groups, or by two-tailed unpaired Student's *t*-test for pairwise comparisons, as indicated. *P* < 0.05 was considered significant.

## SUPPLEMENTARY MATERIAL

Supplementary Material is available at *HMG* online.

## FUNDING

This work was supported by funding from the Swiss National Science Foundation (Grant Nos. 310030_127478 and 31003A_144063 to D.J.M.), the Ecole Polytechnique Fédérale de Lausanne (D.J.M.) and the Michael J. Fox Foundation for Parkinson's Research (D.J.M. and R.B.). This work was supported in part by grants from the NIH NS38377 (Y.X., V.L.D. and T.M.D.). T.M.D. is the Leonard and Madlyn Abramson Professor in Neurodegenerative Diseases. Y.X., V.L.D. and T.M.D. acknowledge the joint participation by the Adrienne Helis Malvin Medical Research Foundation through its direct engagement in the continuous active conduct of medical research in conjunction with The Johns Hopkins Hospital and the Johns Hopkins University School of Medicine and the Foundation's Parkinson's Disease Programs.

## Supplementary Material

Supplementary Data
