# Supplementary material for: Functional interaction of Parkinson's disease-associated LRRK2 with members of the dynamin GTPase superfamily
Source: Hum Mol Genet. 2013 Nov 26;23(8):2055–77. doi: 10.1093/hmg/ddt600 (PMC3959816; doi:10.1093/hmg/ddt600)
Supplement: Supplementary Data [file supp_23_8_2055__index.html]

Functional interaction of Parkinson's disease-associated LRRK2 with members of the dynamin GTPase superfamily — Functional interaction of Parkinson's disease-associated LRRK2 with members of the dynamin GTPase superfamily — Supplementary Data 

# Functional interaction of Parkinson's disease-associated LRRK2 with members of the dynamin GTPase superfamily

## Supplementary Data

Supplementary Data

**Files in this Data Supplement:**

- Supplementary Data - Pdf file
