# Supplementary material for: Functional interaction of Parkinson's disease-associated LRRK2 with members of the dynamin GTPase superfamily
Source: Hum Mol Genet. 2013 Nov 26;23(8):2055–77. doi: 10.1093/hmg/ddt600 (PMC3959816; doi:10.1093/hmg/ddt600)
Supplement: Supplementary Data [file supp_ddt600_ddt600supp.pdf]

Supplementary data

Figure S1. *LRRK2 interacts with OPA1.* (**A**) Domain mapping reveals the interaction of Myc-tagged L-OPA1 with full-length (WT) LRRK2 but isolated domains of LRRK2 following IP with anti-FLAG antibody from HEK-293T cells. Domain organization of LRRK2 deletion mutants is indicated. (**B**) Myc-tagged L-OPA1 interacts with WT and PD-associated mutant forms of FLAG-LRRK2 following IP with anti-FLAG antibody from HEK-293T cells. No differences are observed in the interactions of L-OPA1 with R1441C, Y1699C and G2019S LRRK2 compared to WT LRRK2. Blots are representative of at least two independent experiments.

Figure S2. *LRRK2 partially co-localizes with early and late endosomes in cortical neurons.* Confocal fluorescence microscopy reveals the partial co-localization of FLAG-tagged human LRRK2 variants (WT, R1441C or G2019S) with RFP-Rab5-positive and GFP-Rab7-positive early and late endosomes, respectively, in rat primary cortical neurons. Cytofluorograms and co-localization coefficients (Rcoloc; mean ± SEM, $n \geq 5$ cells) reveal the extent of co-localization of LRRK2 with Rab5 and Rab7 fluorescence signals. LRRK2 preferentially co-localizes with early (Rab5) compared to late (Rab7) endosomes. Confocal images are taken from a single *z*-plane at 0.1 µm thickness. Images are representative of multiple neurons from at least two independent transfection experiments. Scale bars: 10 µm.

Figure S3. *LRRK2 attenuates neurite shortening induced by dominant-negative K44A Dnm1.* (**A**) Primary cortical neurons were co-transfected with FLAG-tagged WT LRRK2, GFP-tagged K44A Dnm1 and DsRed-Max constructs at a molar ratio of 10:10:1 at DIV 3 and fixed at DIV 7. Analysis of DsRed-positive neurites reveals a marked shorting of axonal processes by K44A Dnm1 expression alone, with a negligible effect of WT LRRK2 expression alone, compared to control neurites (DsRed alone). Co-expression of WT LRRK2 and K44A Dnm1 markedly attenuates the K44A Dnm1-induced shortening of axonal processes. Bars represent axonal processes length (mean ± SEM) expressed as a percent of DsRed alone (control) from ≥90 DsRed-positive neurons taken from at least three independent experiments/cultures. $^{**}P<0.01$ or $^{***}P<0.005$ by one-way ANOVA with Newman-Keuls post-hoc analysis. (**B**) Western blot

analysis with anti-FLAG, anti-GFP and anti-β-tubulin antibodies of cell extracts derived from rat primary cortical neurons at DIV 7 transiently expressing FLAG-LRRK2 and K44A Dnm1-GFP. Densitometric analysis reveals a significant reduction of K44A Dnm1 levels in the presence of LRRK2, and a significant reduction of LRRK2 in the presence of K44A Dnm1. Graphs indicate LRRK2 (*left*) or K44A Dnm1 (*right*) steady-state levels normalized to β-tubulin levels, expressed as a percent of each protein alone (mean ± SEM, n = 3 experiments). **P<0.01 or ***P<0.005 by unpaired Student's t-test, as indicated. *ns*, non-significant.

Figure S4. *Effect of LRRK2 on Dnm1 protein solubility, degradation and turnover in neurons*. (**A**) Western blot analysis with anti-GFP, anti-FLAG and anti-β-tubulin antibodies of Triton-soluble and Triton-insoluble (RIPA-soluble) fractions derived from rat primary cortical neurons at DIV 7 expressing Dnm1-GFP and FLAG-LRRK2. Cells were treated for 24 h prior to harvesting with the proteasome inhibitor, MG132 (5 µM), or the lysosome inhibitor, bafilomycin A1 (50 nM). LRRK2 expression decreases Dnm1 levels in the Triton-soluble fraction, but Dnm1 levels are not recovered by MG132 or bafilomycin A1 treatment, nor does Dnm1 accumulate in the Triton-insoluble fraction in the presence of LRRK2. Blots are representative of at least independent experiments. (**B**) Primary cortical neurons expressing Dnm1-GFP with or without FLAG-LRRK2 were treated with cycloheximide (CHX, 100 µg/ml) at DIV 7, and cultures were harvested at 0 (untreated control), 1, 2, 3, 6, 12 and 24 h post-treatment. Western blot analysis was conducted on soluble extracts from cortical neurons with anti-GFP antibody to monitor Dnm1 turnover, or with anti-β-tubulin antibody to demonstrate equal protein loading. Densitometric analysis indicates the equivalent turnover of Dnm1 in the presence or absence of LRRK2. Dnm1 levels were normalized to β-tubulin levels and expressed as a percent of time point '0' for each transfection condition and time point (mean ± SEM; n = 3 experiments).

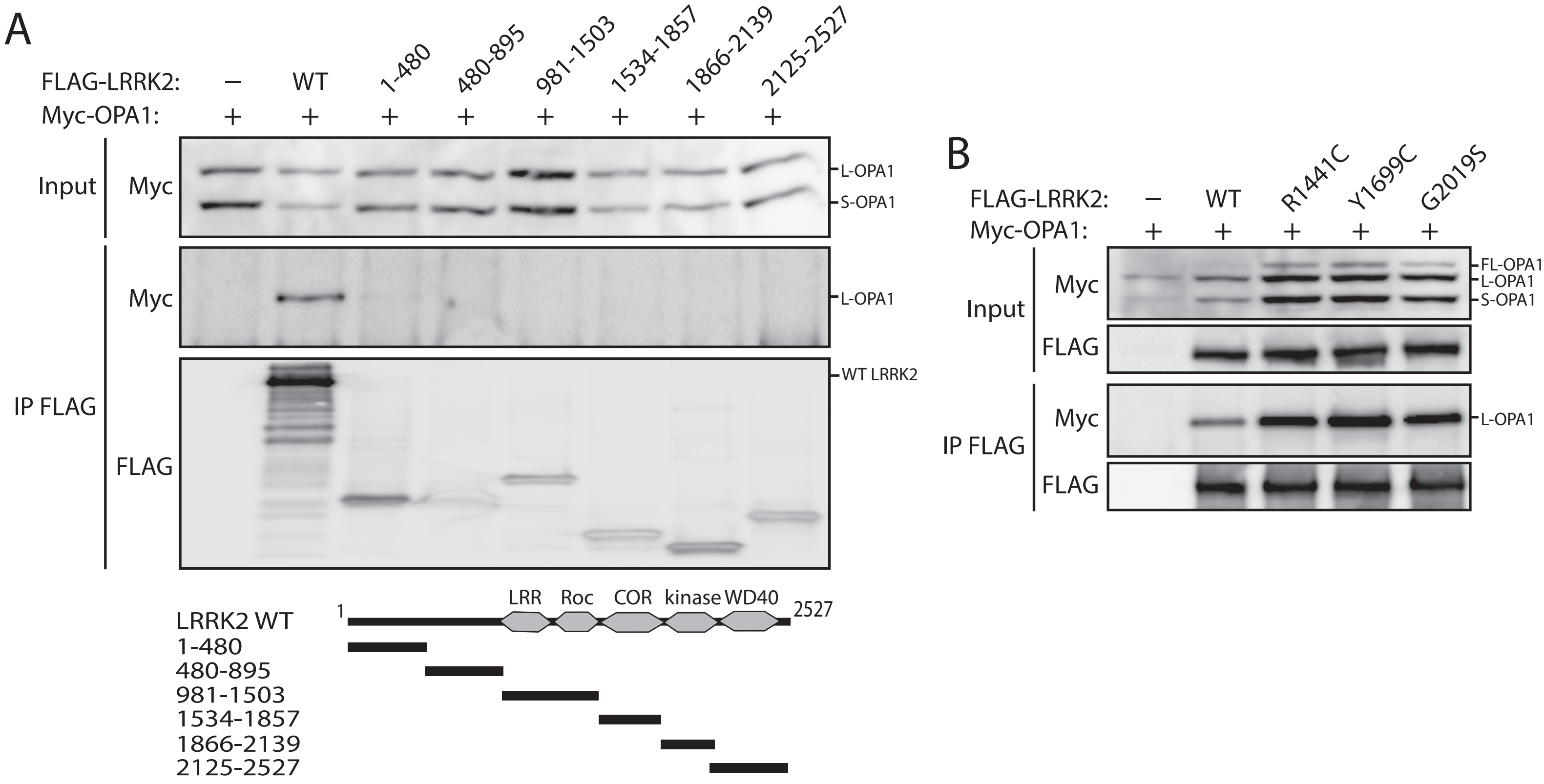
A
FLAG-LRRK2:
—
WT
1-480
480-895
981-1503
1534-1857
1866-2139
2125-2527
Myc-OPA1:
+
Input
Myc
L-OPA1
S-OPA1
IP FLAG
Myc
L-OPA1
FLAG
WT LRRK2
LRRK2 WT
1
LRR
Roc
COR
kinase
WD40
2527
1-480
480-895
981-1503
1534-1857
1866-2139
2125-2527
B
FLAG-LRRK2:
—
WT
R1441C
Y1699C
G2019S
Myc-OPA1:
+
Input
Myc
FL-OPA1
L-OPA1
S-OPA1
FLAG
IP FLAG
Myc
L-OPA1
FLAG

Figure S1

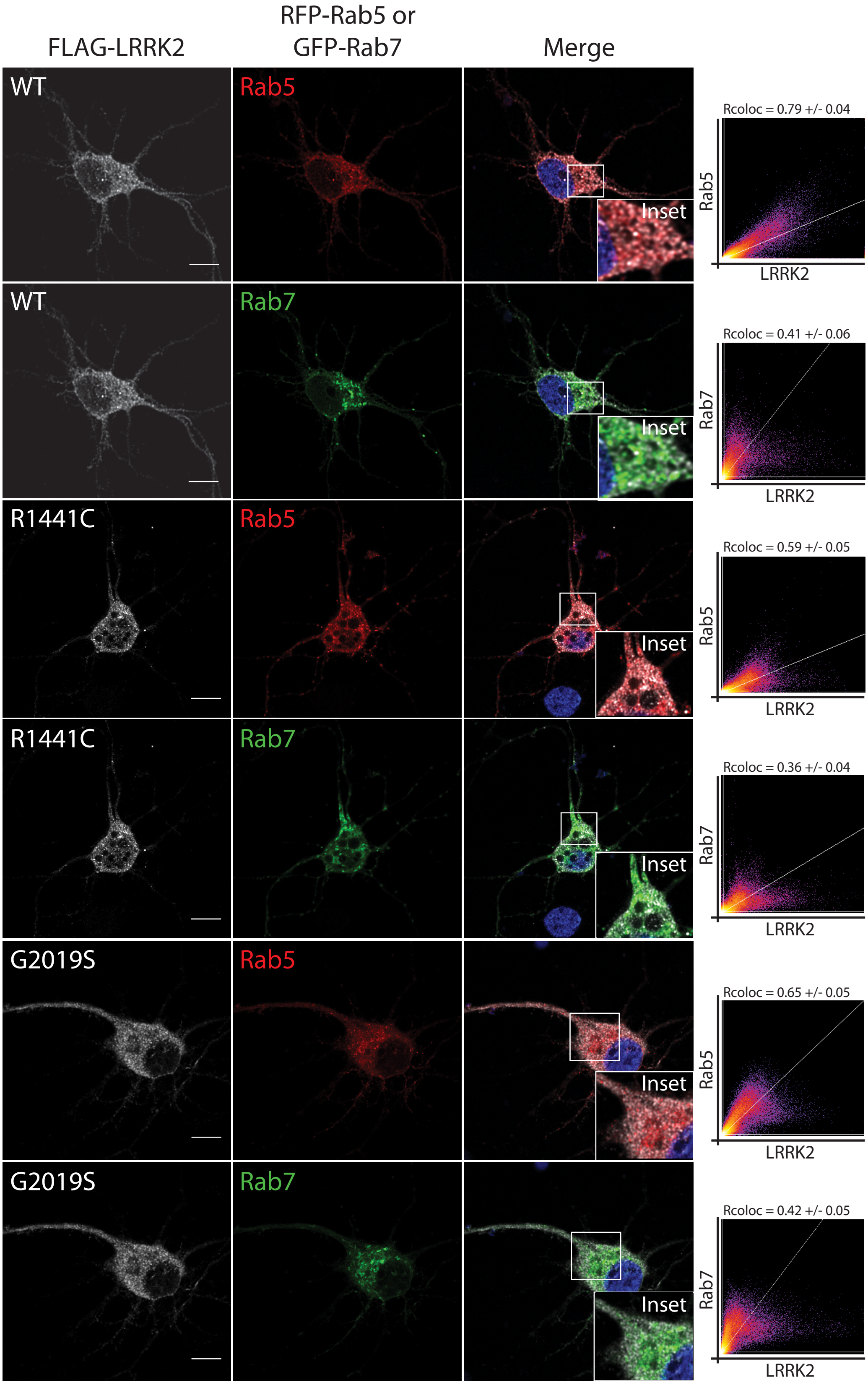
FLAG-LRRK2
RFP-Rab5 or GFP-Rab7
Merge
WT
Rab5
Inset
Rcoloc = 0.79 +/- 0.04
Rab5
LRRK2
WT
Rab7
Inset
Rcoloc = 0.41 +/- 0.06
Rab7
LRRK2
R1441C
Rab5
Inset
Rcoloc = 0.59 +/- 0.05
Rab5
LRRK2
R1441C
Rab7
Inset
Rcoloc = 0.36 +/- 0.04
Rab7
LRRK2
G2019S
Rab5
Inset
Rcoloc = 0.65 +/- 0.05
Rab5
LRRK2
G2019S
Rab7
Inset
Rcoloc = 0.42 +/- 0.05
Rab7
LRRK2

Figure S2

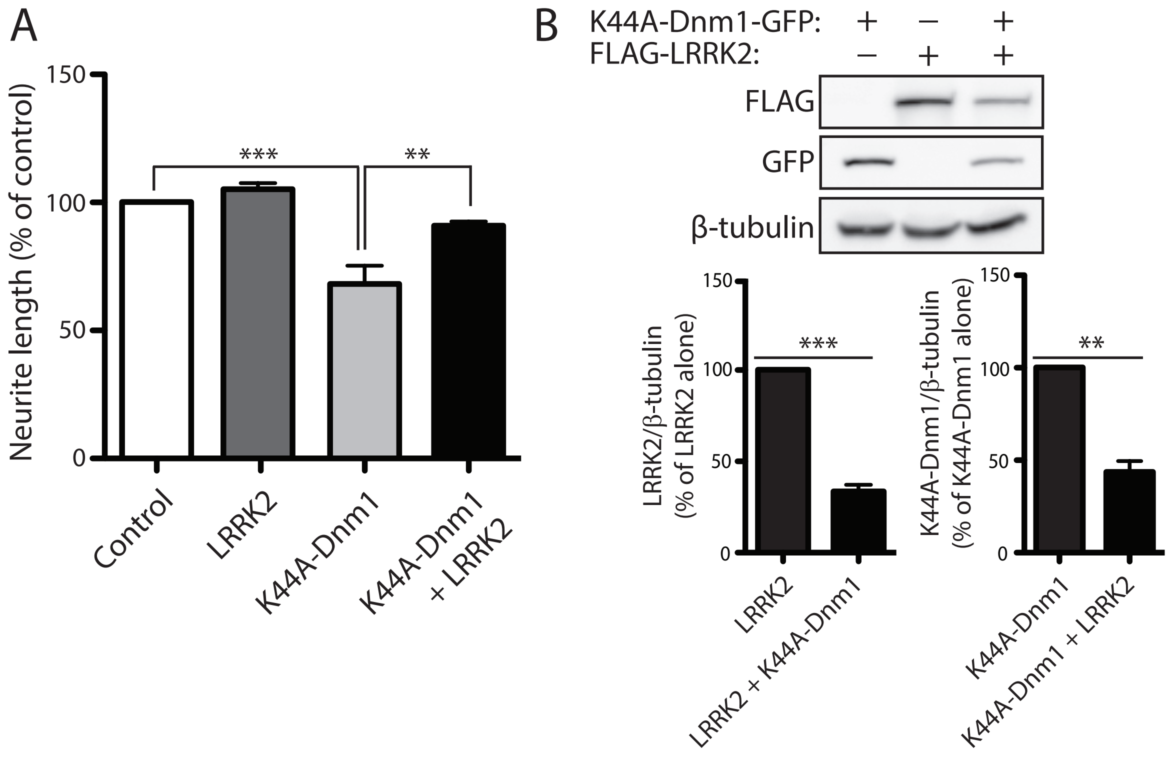
A
Neurite length (% of control)
150
100
50
0
***
**
Control
LRRK2
K44A-Dnm1
K44A-Dnm1 + LRRK2
B
K44A-Dnm1-GFP: + − +
FLAG-LRRK2: − + +
FLAG
GFP
β-tubulin
LRRK2/β-tubulin (% of LRRK2 alone)
***
LRRK2
LRRK2 + K44A-Dnm1
K44A-Dnm1/β-tubulin (% of K44A-Dnm1 alone)
**
K44A-Dnm1
K44A-Dnm1 + LRRK2

Figure S3

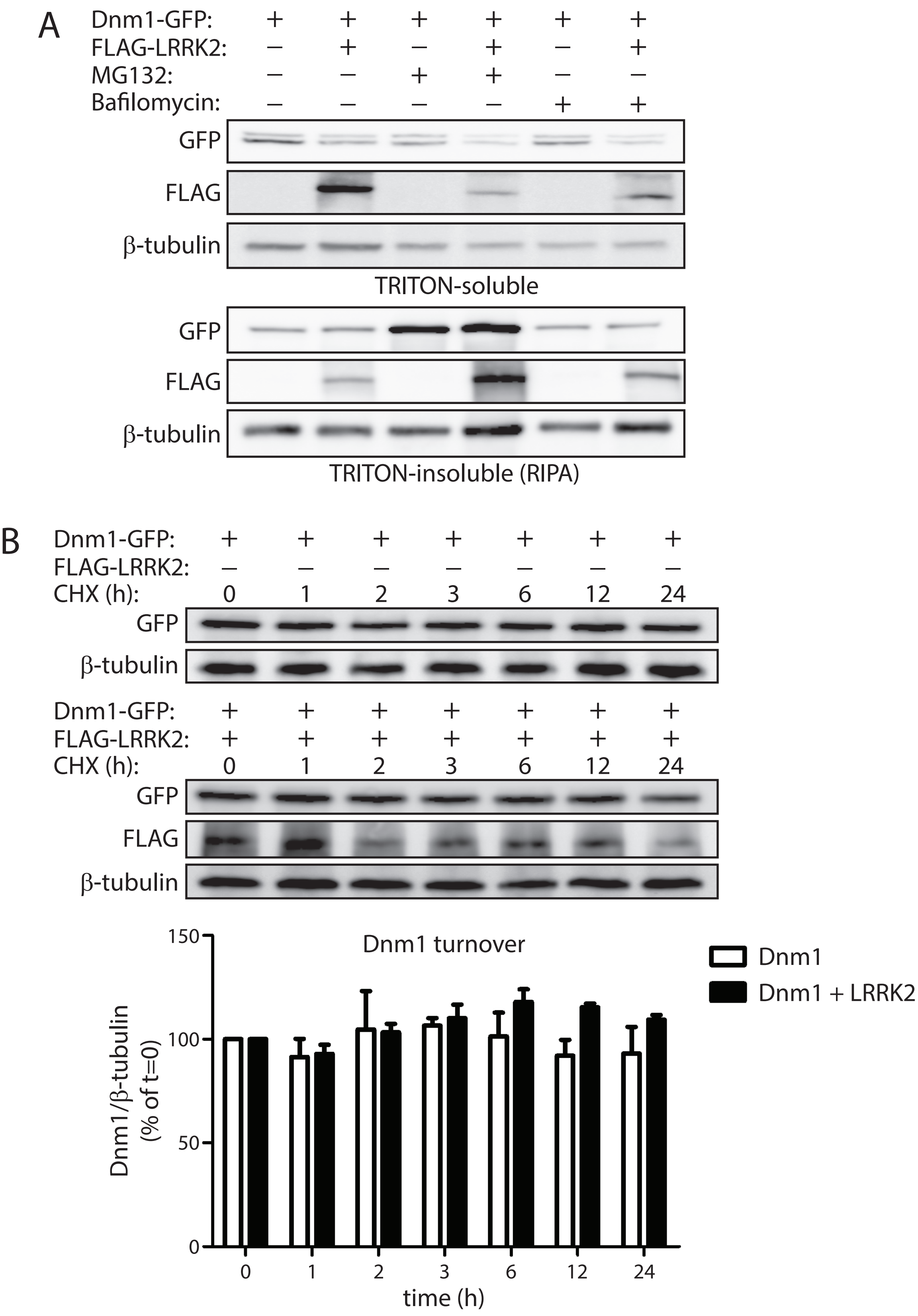
A
Dnm1-GFP:
FLAG-LRRK2:
MG132:
Bafilomycin:
GFP
FLAG
β-tubulin
TRITON-soluble
GFP
FLAG
β-tubulin
TRITON-insoluble (RIPA)
B
Dnm1-GFP:
FLAG-LRRK2:
CHX (h):
0
1
2
3
6
12
24
GFP
β-tubulin
Dnm1-GFP:
FLAG-LRRK2:
CHX (h):
GFP
FLAG
β-tubulin
Dnm1 turnover
Dnm1
Dnm1 + LRRK2
Dnm1/β-tubulin
(% of t=0)
150
100
50
0
time (h)

Figure S4
